# Dosimetric evaluation of the compass program for patient dose analysis in IMRT delivery quality assurance

**DOI:** 10.1371/journal.pone.0209180

**Published:** 2018-12-20

**Authors:** Ju-Young Song, Sung-Ja Ahn

**Affiliations:** Department of Radiation Oncology, Chonnam National University Medical School, Gwangju, Korea; North Shore Long Island Jewish Health System, UNITED STATES

## Abstract

A practical method was designed to verify the accuracy of dose distributions calculated using Compass, which can reconstruct the dose distribution inside a patient’s body during intensity-modulated radiation therapy (IMRT). Twelve virtual IMRT treatment plans were developed using an ArcCHECK diode detector array, and then the recalculated and reconstructed doses in Compass were compared with the actual measurements to assess the dosimetric accuracy. Based on the results of gamma evaluation for the 12 plans, Compass achieved average pass rates higher than 98%, which confirmed proper dosimetric accuracy in the IMRT quality assurance process. The validity of Compass for clinical applications was also confirmed through an additional comparison with the results calculated using 3DVH, another dose reconstruction program. It is necessary to verify the accuracy of the dose calculated using the program in advance before the commercialized dose reconstruction program is applied in clinical practice. This study has limitations in that it did not provide a real scientific contribution such as an introduction of new algorithm for dose calculation and the development of new measurement tools. However, the method based on the comparative analysis with the actual measured dose values as devised in this study seems to be useful in that it can be applied effectively to verify the dosimetric accuracy of the dose reconstruction program before first using it in the clinical cases.

## Introduction

Delivery quality assurance (DQA) has been investigated in various studies for the verification of the dosimetric accuracy of intensity-modulated radiation therapy (IMRT) and volumetric modulated arc therapy (VMAT) [1–5]. A conventional procedure used for IMRT DQA is measurement of a dose distribution in a phantom structure. The dosimetric errors are thereafter analyzed by comparing the measured data with the calculated dose in a treatment planning system (TPS).

The conventional DQA process has some limitations because it measures and analyzes the dose in a phantom material and not within the body of the patient [6–8]. In order to overcome this limitation, special tools were developed for calculating the dose distribution in a patient’s body using the measured data in the DQA process [9,10]. Typical tools that employ this method include Compass (IBA, Schwarzenbruck, Germany), 3DVH (SunNuclear, Melbourne, FL, USA), and Delta4DVH (ScandiDos, Uppsala, Sweden) [11–15]. Compass calculates the dose inside a patient’s body using two different methods. One method is the calculation-based TPS check, which recalculates the dose using a TPS class collapsed cone algorithm without the phantom measurement. The other method is the dose reconstruction based on the measured data using a two-dimensional (2D) detector array such as MatriXX (IBA, Schwarzenbruck, Germany) or Dolphin (IBA, Schwarzenbruck, Germany).

Although the dosimetric accuracy of Compass was analyzed in various studies and appropriate accuracy was demonstrated for IMRT and VMAT, most of the studies were limited to verifying the accuracy of the calculated dose by comparing it with the dose distribution calculated using other reference dose calculation tools such as a Monte Carlo simulation [16–18]. In addition, few studies have been conducted for the analysis of dose accuracy calculated using Compass through the actual measurement of dose distribution for various clinical cases. In order to apply a dose recalculation tool such as Compass to clinical cases, the confirmation of tool accuracy and characteristics of the calculated dose should be performed by comparing the actual measured dose distribution for various cases with the dose distribution calculated using the tool.

In this study, a practical method was developed to verify the accuracy and characteristics of dose distributions calculated using Compass through a comparative analysis with the actual measured dose values before applying Compass to clinical practice.

After acquiring computed tomography (CT) images of ArcCHECK (SunNuclear, Melbourne, FL, USA) for IMRT DQA, IMRT plans were made based on the virtual tumor targets and organs at risk (OARs), which were contoured on the CT images, for typical clinical cases. The dosimetric accuracy of Compass was evaluated by analyzing the degree of similarity between the dose distribution by the actual beam delivery measured at the detector array of ArcCHECK, the recalculated dose distribution in Compass, and the reconstructed dose distribution based on the data measured using MatriXX.

In addition, we evaluated the dosimetric characteristics of Compass by comparing the calculated dosimetric results in the typical cancer cases with the results calculated using another dose reconstruction program, 3DVH.

## Materials and methods

### Verification of the calculated dose accuracy in compass

#### Preparation of the virtual treatment plans

**Fig 1:**
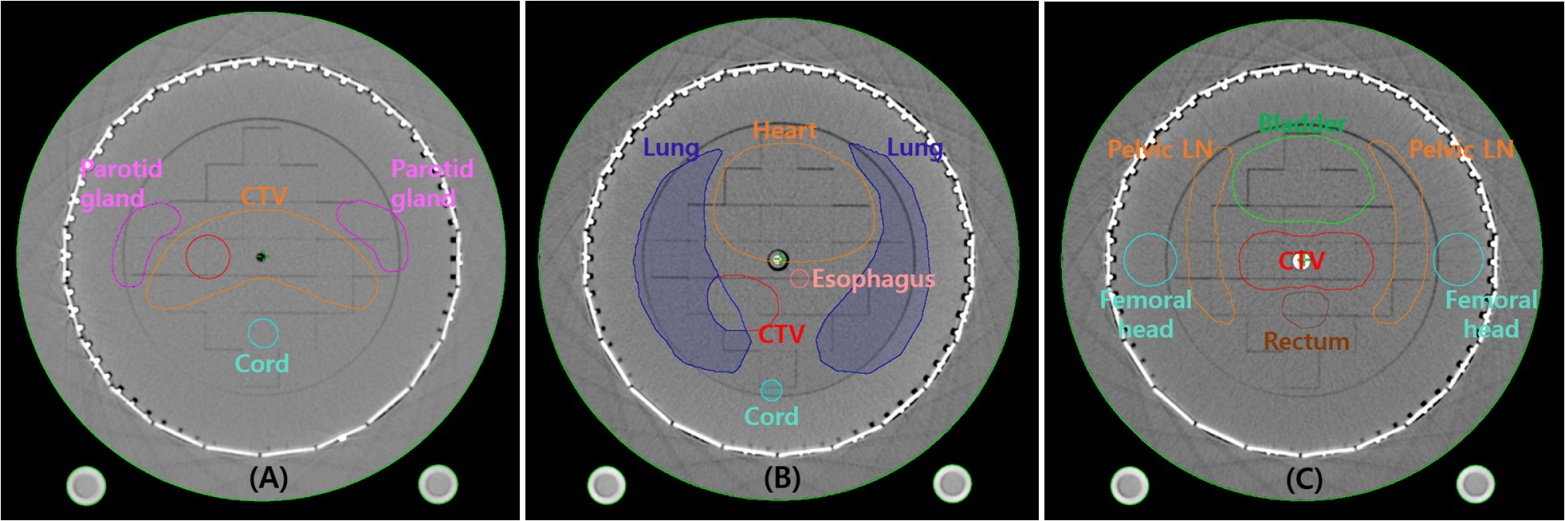
Contours of tumor target and OARs delineated on the ArcCHECK phantom. (A) head-neck plan, (B) lung plan, (C) prostate plan.

The diode detector array ArcCHECK was used for measurement of the real dose distribution of IMRT and VMAT. After acquiring a CT image of the ArcCHECK, virtual treatment plans were prepared for head-neck, lung and prostate cases based on the CT images. In each cancer cases, the IMRT and RapidArc (Varian, Palo Alto, CA, USA) plans were made using photons of 6 MV and 10 MV, respectively, and 12 plans were prepared. The virtual tumor target and OARs were contoured as shown in Fig 1. All of the plans were created using an Eclipse (Varian, Palo Alto, CA, USA) planning system and prepared according to the dose prescription, as shown in Table 1. A clinical linear accelerator (LINAC), Trilogy Tx (Varian, Palo Alto, CA, USA), was used in this study.

**Table 1:** Dose constraints used for the IMRT and rapidArc planning.

Head-Neck Plan	
GTV	V_220 cGy_ > 95%
CTV	V_180 cGy_ > 95%
Parotid gland	D_mean_ < 65 cGy
Spinal cord	D_max_ < 110 cGy
Thyroid gland	D_mean_ < 65 cGy
Prostate Plan	
CTV	V_220 cGy_ > 95%
Pelvic lymph node	V_180 cGy_ > 95%
Bladder	D_max_ < 210 cGy, V_140 cGy_ < 40%
Rectum	D_max_ < 210 cGy, V_140 cGy_ < 40%
Femoral head	D_max_ < 120 cGy, V_90 cGy_ < 20%
Lung Plan	
CTV	V_200 cGy_ > 95%
Esophagus	D_max_ < 170 cGy
Spinal cord	D_max_ < 140 cGy
Heart	V_30 cGy_ < 15%
Lung	D_mean_ < 30 cGy

### Dose recalculation with compass

The dose distribution within a patient’s body was recalculated for all of the prepared treatment plans using the two calculation methods in Compass.

**Fig 2:**
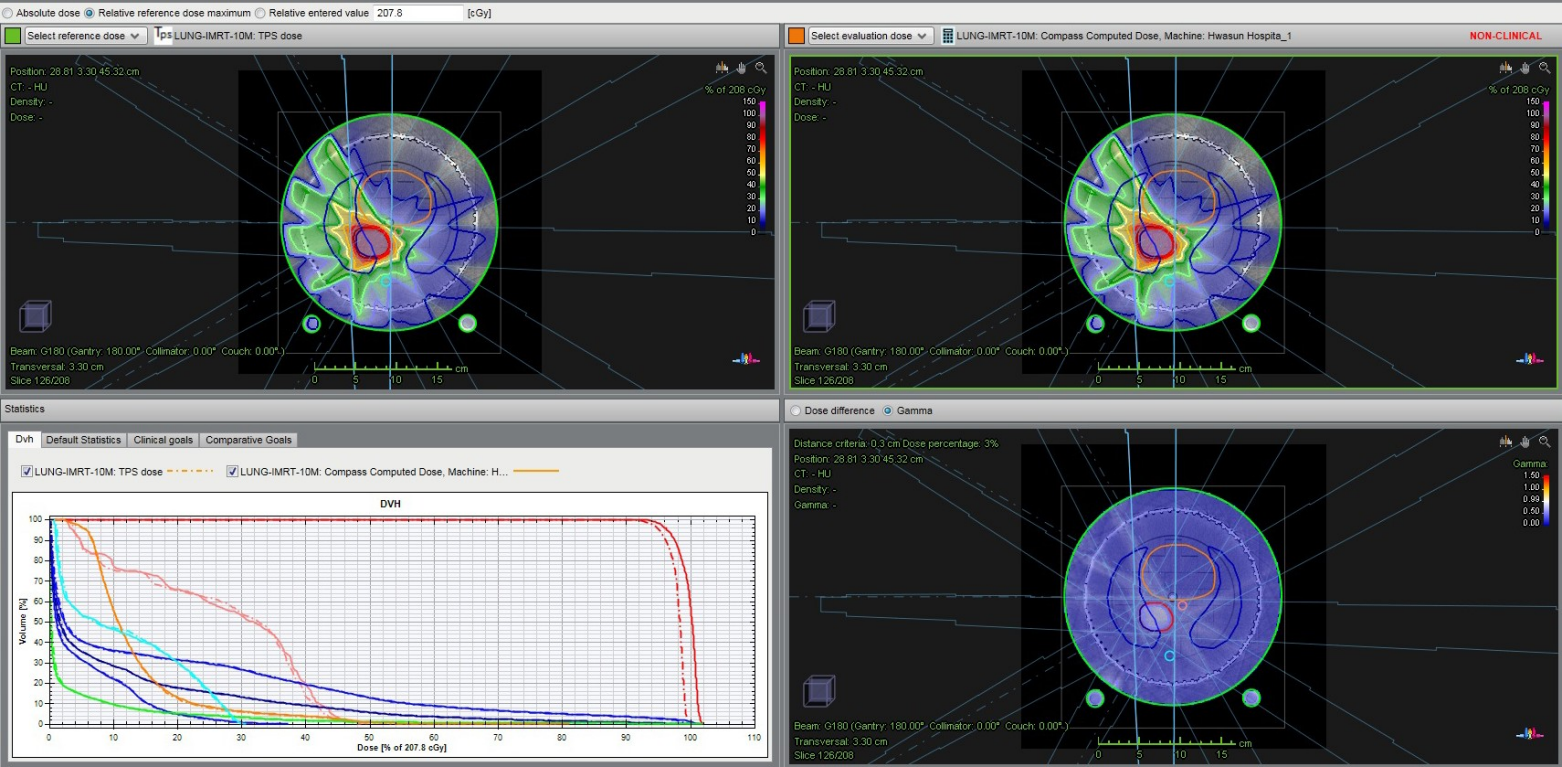
Example of the result of the recalculated dose distribution using the compass program.

First, the plan data including the CT, structures, and plan files created using TPS were imported into Compass in the format of digital imaging and communications in medicine (DICOM), and the dose was recalculated using the collapsed cone algorithm established through the beam commissioning process. Fig 2 shows an example of the results of the recalculated dose distribution.

**Fig 3:**
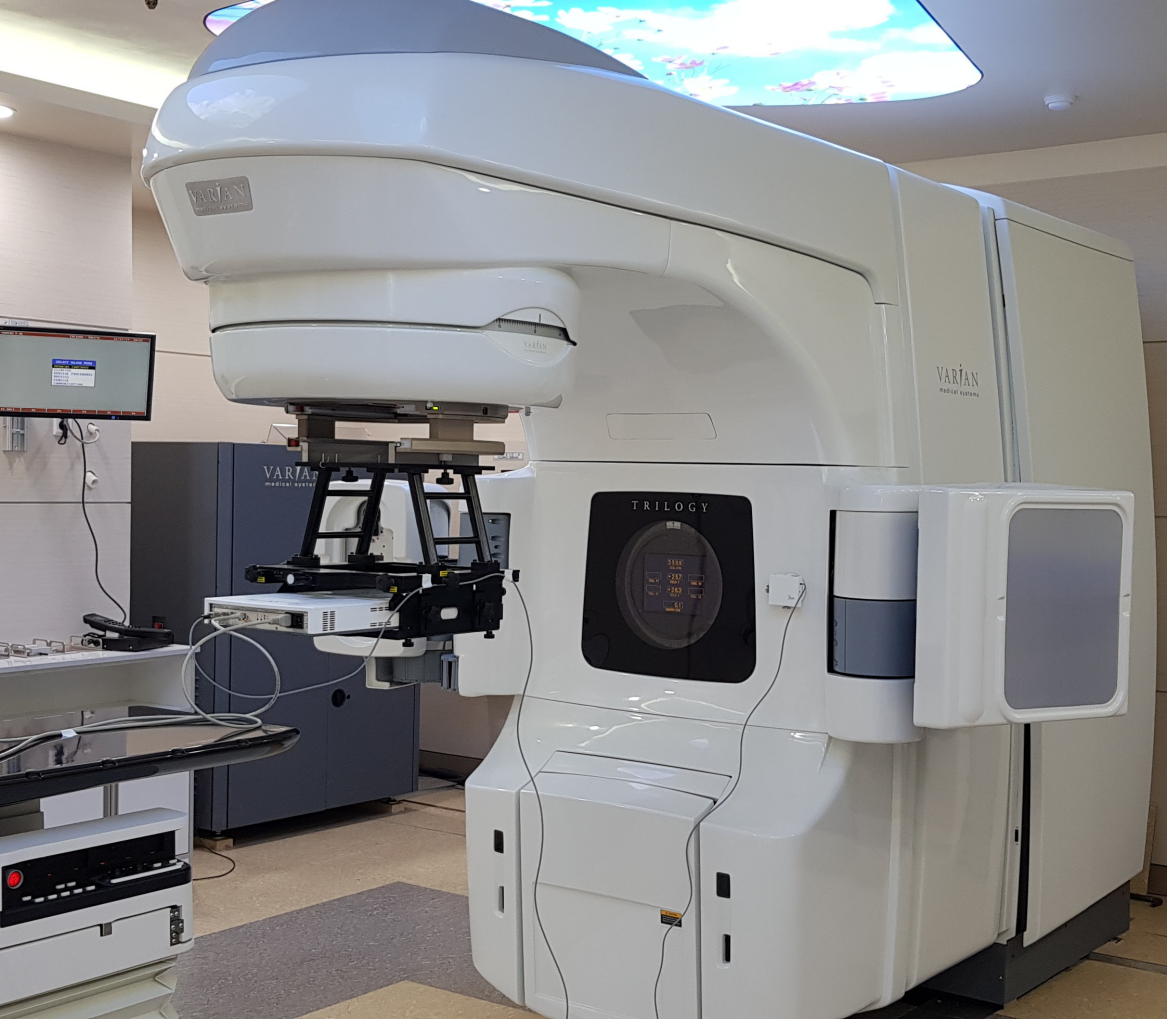
Dose measurement with MatriXX through the actual beam delivery of each plan.

**Fig 4:**
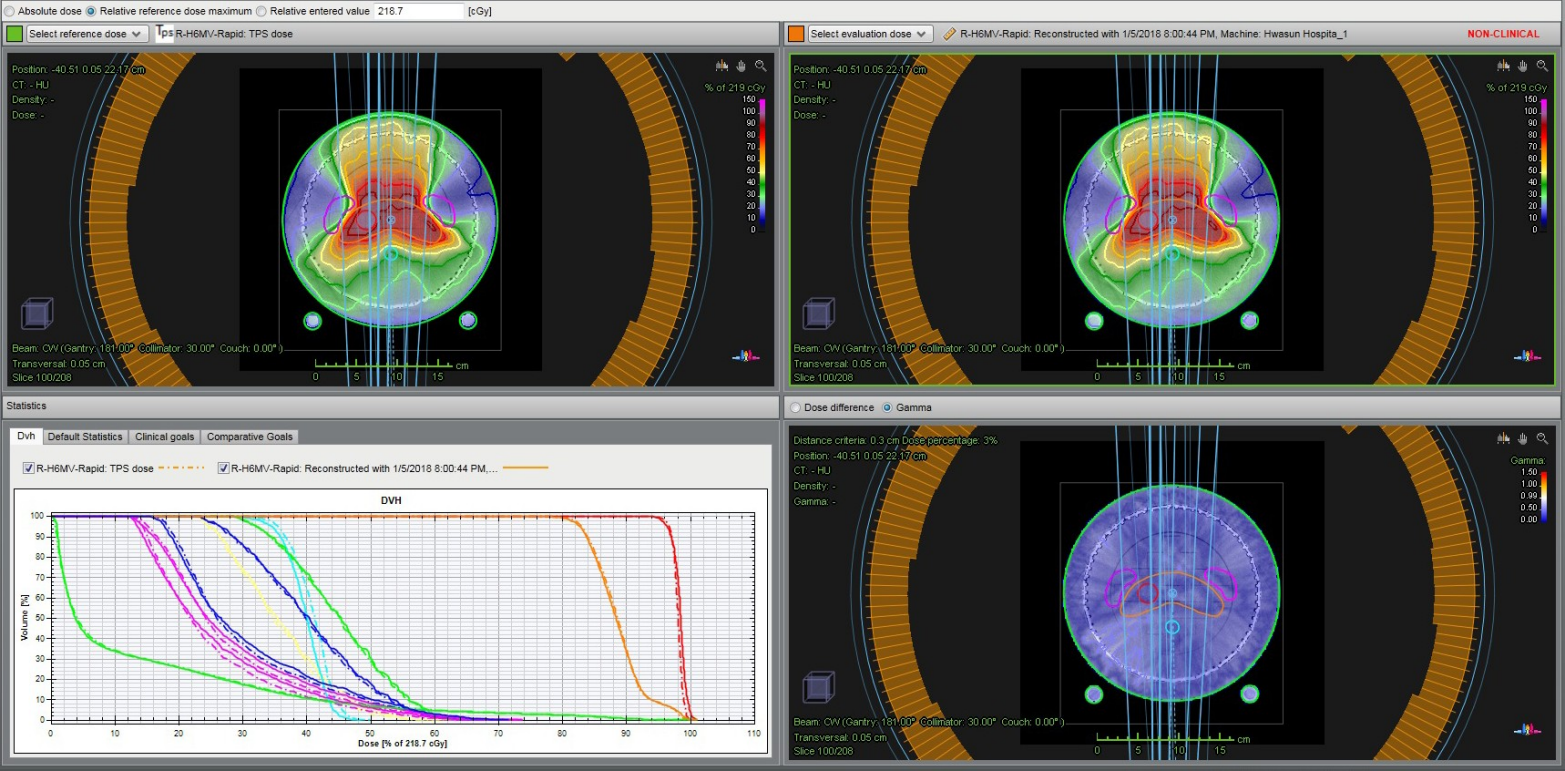
Example of the dose reconstruction based on the dose data measured using MatriXX.

Subsequently, the dose in the patient was reconstructed based on the dose data measured using a 2D ion chamber array MatriXX. Fig 3 shows the process of measuring the dose through actual beam delivery of each plan using MatriXX, and Fig 4 shows an example of dose reconstruction based on the dose data measured using MatriXX.

#### Analysis of the dosimetric accuracy of compass

The dose distribution was measured at the detector array after beam delivery to ArcCHECK in order to verify the dose accuracy calculated using Compass. The dosimetric accuracy was evaluated by comparison of the measured dose distribution with that calculated using Compass at the same position. The dose results calculated using Eclipse TPS, the Compass recalculation results, and the Compass reconstruction results were compared with the measured dose distributions, and the dose difference was evaluated using the gamma evaluation method with a 3% dose difference and a 3-mm distance-to-agreement criteria. The ArcCHECK tool used in this study was introduced three years ago and the accuracy of dose measurements was verified by commissioning procedure before using it in clinical cases. And it can be evaluated as a reliable tool through the DQA results of VMAT and IMRT so far.

### Comparison of the results obtained from compass and 3DVH

In order to analyze the additional characteristics of the dose distribution calculated using Compass, we performed a comparative evaluation with the results calculated using the 3DVH program which can reconstruct the dose based on the data measured in the DQA process using ArcCHECK. The dose calculation results obtained from Compass and 3DVH were compared and analyzed with 30 RapidArc plans (10 head-neck plans, 10 lung plans, and 10 prostate plans).

**Fig 5:**
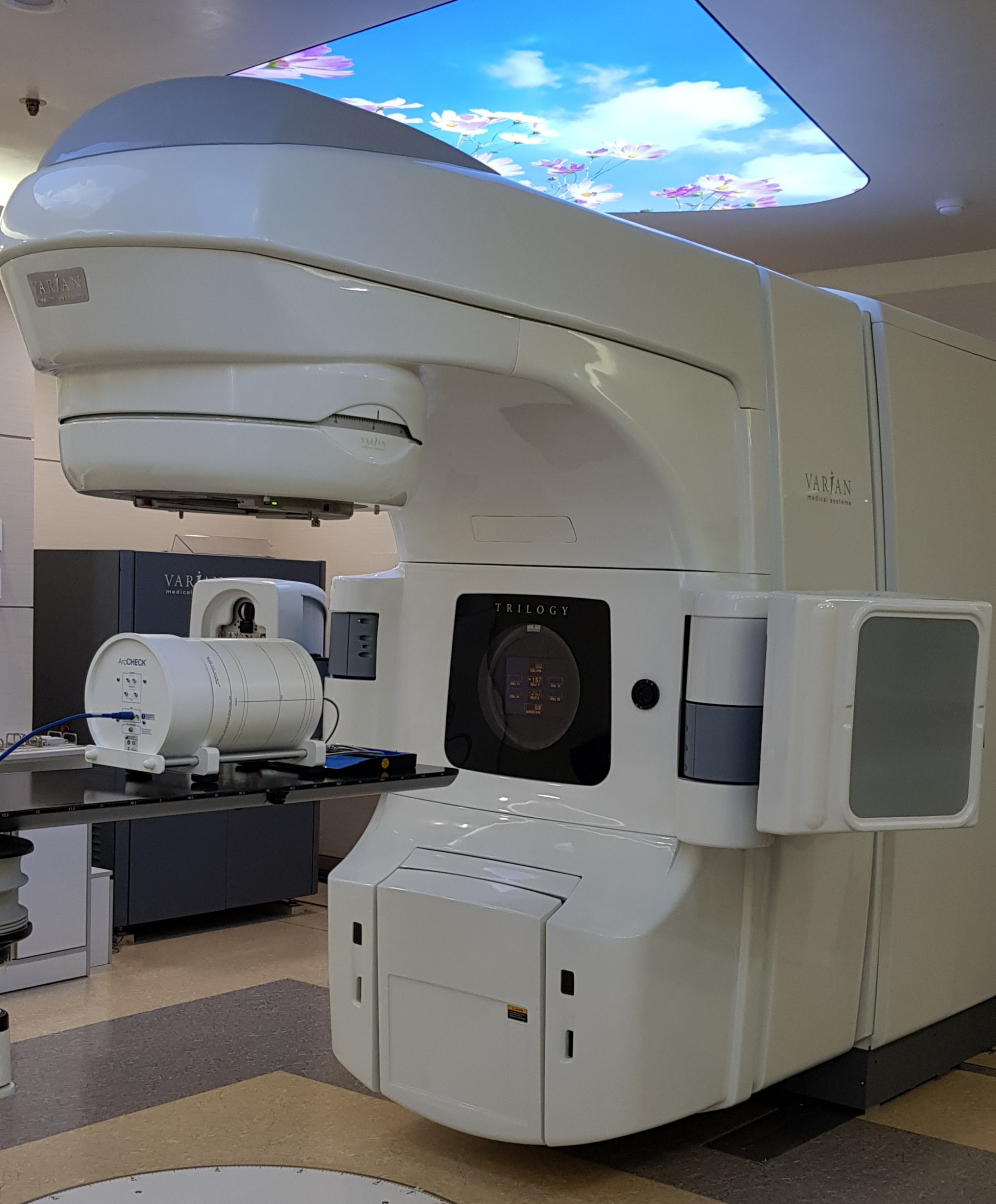
Dose measurement with ArcCHECK for the dose reconstruction with the 3DVH program.

The patient dose distribution in 3DVH was calculated based on the actual measured data by delivering the DQA plan using ArcCHECK as shown in Fig 5. Compass was also used to reconstruct the patient dose for the same plans. The dose distribution recalculated based on the plan data imported from Eclipse TPS was obtained and the reconstructed dose distribution results based on the data measured using MatriXX were also obtained. To evaluate the similarity between the calculated results of the three dose distributions and the results calculated using Eclipse TPS, the pass rates of the gamma evaluation in the tumor target and OARs were calculated with a 3% dose difference and 3-mm distance-to-agreement criteria. In addition, we evaluated the variation in the major dose metrics of the tumor target and OARs according to the results of the three dose calculations.

## Results

**Fig 6:**
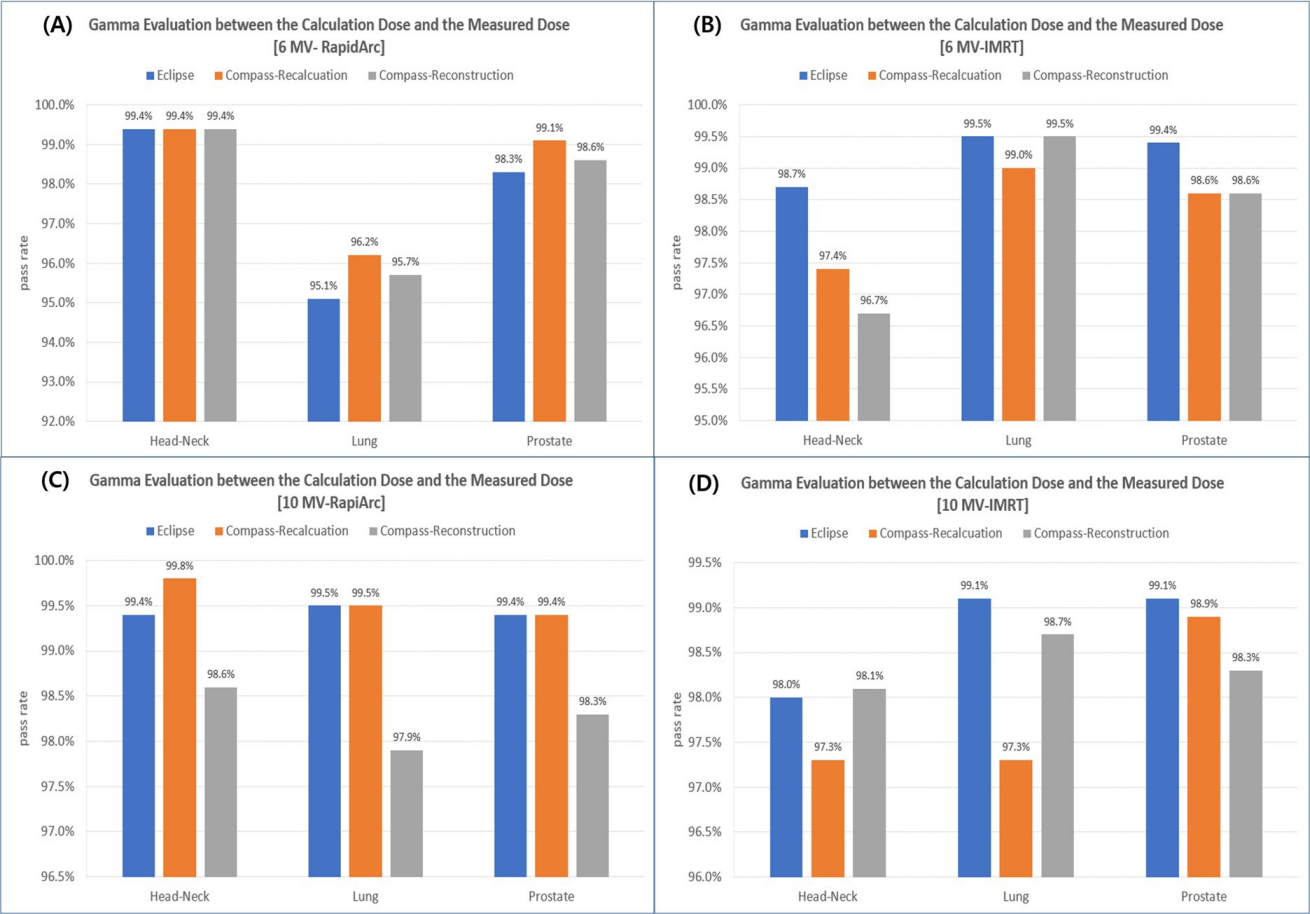
Comparison of the pass rate calculated in a gamma evaluation between a measured dose using ArcCHECK and a predicted dose for each dose calculation tool. (A) 6 MV-RapidArc plans, (B) 6 MV-IMRT plans, (C) 10 MV-RapidArc plans, (D) 10 MV-IMRT plans.

The gamma evaluation results from the comparison with the dose distribution measured using ArcCHECK to verify the accuracy of the recalculated dose and reconstructed doses in Compass are shown in Fig 6.

The average pass rates according to the dose calculation methods were calculated as 98.7±0.8% in Eclipse TPS, 98.5±0.8% in Compass recalculation and 98.2±0.2% in Compass reconstruction. This confirms that the Compass calculation results are similar to the doses calculated using the Eclipse TPS and are accurate without any significant errors compared with the actual measurements.

The average pass rate results according to the photon energy, treatment technique, and treatment legion showed similar accuracy without any significant differences, as shown in Table 2.

**Table 2:** Average pass rate calculated in a gamma evaluation between a measured dose using ArcCHECK and a predicted dose in each of the dose calculation tools.

	Eclipse	Compass-Recalculation	Compass-Reconstruction
6MV	98.4±1.7%	98.3±1.2%	98.1±1.5%
10MV	99.1±0.6%	98.7±1.1%	98.3±0.3%
IMRT	98.9±0.6%	98.1±0.8%	98.3±0.9%
RapidArc	98.5±1.7%	98.9±1.3%	98.1±1.3%
Head-Neck	98.9±0.7%	98.5±1.3%	98.2±1.1%
Lung	98.3±2.1%	98.0±1.5%	97.9±1.6%
Prostate	99.1±0.5%	99.0±0.3%	98.5±0.2%

The results of the comparative evaluation with the dose calculated using the 3DVH program are shown in Tables 3–8. Tables 3–5 show the pass rates of the gamma evaluation in the tumor target and OARs when the calculation results obtained from Compass and 3DVH were compared with the reference dose distribution calculated using Eclipse TPS.

**Table 3:** Pass rates calculated for the gamma evaluation in the tumor target and OARs compared with a reference TPS dose with the calculated dose in 3DVH and compass (C-Recal.: Compass Recalculation, C-Recon.: Compass Reconstruction) for the Head-Neck plans.

		CTV	Brain Stem	Eyeball	Cord	Parotid Gland	Eso-phagus	Thyroid	Hippo-campus
H1	3DVH	97.6%	100.0%	100.0%					
	C-Recal.	100.0%	100.0%	100.0%					
	C-Recon.	100.0%	100.0%	100.0%					
H2	3DVH	98.2%	99.7%		99.5%	100.0%	99.1%		
	C-Recal.	99.5%	100.0%		100.0%	100.0%	99.8%		
	C-Recon.	95.8%	100.0%		100.0%	99.9%	99.5%		
H3	3DVH	99.7%	100.0%		100.0%	100.0%	100.0%		
	C-Recal.	99.8%	100.0%		100.0%	100.0%	100.0%		
	C-Recon.	97.1%	100.0%		100.0%	100.0%	100.0%		
H4	3DVH	96.9%			98.3%			97.0%	
	C-Recal.	95.6%			100.0%			100.0%	
	C-Recon.	69.1%			99.5%			93.1%	
H5	3DVH	93.9%	100.0%						100.0%
	C-Recal.	100.0%	100.0%						100.0%
	C-Recon.	92.8%	100.0%						100.0%
H6	3DVH	96.7%	98.5%						
	C-Recal.	100.0%	100.0%						
	C-Recon.	99.3%	96.8%						
H7	3DVH	90.3%	99.1%		97.2%				
	C-Recal.	100.0%	100.0%		100.0%				
	C-Recon.	99.1%	99.5%		100.0%				
H8	3DVH	99.5%	100.0%	100.0%					
	C-Recal.	100.0%	100.0%	100.0%					
	C-Recon.	100.0%	100.0%	100.0%					
H9	3DVH	97.2%			100.0%	100.0%	100.0%		
	C-Recal.	99.2%			100.0%	100.0%	100.0%		
	C-Recon.	91.0%			100.0%	100.0%	100.0%		
H10	3DVH	100.0%	100.0%	100.0%					
	C-Recal.	100.0%	100.0%	100.0%					
	C-Recon.	100.0%	100.0%	100.0%					

**Table 4:** Pass rates calculated for the gamma evaluation in the tumor target and OARs compared with a reference TPS dose with the calculated dose in 3DVH and compass (C-Recal.: Compass Recalculation, C-Recon.: Compass Reconstruction) for the lung plans.

		CTV	Lung	Heart	Cord	Esophagus
L1	3DVH	100.0%	100.0%	100.0%	100.0%	100.0%
	C-Recal.	99.9%	99.9%	100.0%	100.0%	100.0%
	C-Recon.	97.8%	99.8%	100.0%	100.0%	100.0%
L2	3DVH	86.3%	99.8%	100.0%	100.0%	100.0%
	C-Recal.	99.6%	100.0%	100.0%	100.0%	100.0%
	C-Recon.	81.9%	100.0%	100.0%	100.0%	99.5%
L3	3DVH	99.7%	99.8%	100.0%	100.0%	100.0%
	C-Recal.	98.9%	99.7%	100.0%	100.0%	100.0%
	C-Recon.	94.5%	99.5%	100.0%	100.0%	100.0%
L4	3DVH	99.7%	100.0%	100.0%	100.0%	99.6%
	C-Recal.	99.1%	99.9%	100.0%	100.0%	99.6%
	C-Recon.	97.1%	99.8%	99.90%	100.0%	100.0%
L5	3DVH	99.1%	99.8%	100.0%	100.0%	99.9%
	C-Recal.	98.2%	99.9%	100.0%	100.0%	99.5%
	C-Recon.	97.5%	99.8%	100.0%	100.0%	99.8%
L6	3DVH	97.7%	99.9%	100.0%	100.0%	100.0%
	C-Recal.	99.6%	100.0%	100.0%	100.0%	100.0%
	C-Recon.	98.0%	99.9%	100.0%	100.0%	99.9%
L7	3DVH	92.7%	100.0%	100.0%	100.0%	100.0%
	C-Recal.	100.0%	100.0%	100.0%	100.0%	100.0%
	C-Recon.	91.1%	100.0%	100.0%	100.0%	100.0%
L8	3DVH	98.9%	99.8%	100.0%	100.0%	100.0%
	C-Recal.	99.6%	100.0%	100.0%	100.0%	100.0%
	C-Recon.	99.2%	99.9%	100.0%	100.0%	100.0%
L9	3DVH	99.7%	100.0%	100.0%	100.0%	100.0%
	C-Recal.	96.9%	99.1%	100.0%	99.4%	99.9%
	C-Recon.	99.9%	99.8%	100.0%	99.9%	100.0%
L10	3DVH	99.6%	99.9%	99.8%	100.0%	99.9%
	C-Recal.	99.2%	96.8%	100.0%	100.0%	99.4%
	C-Recon.	97.7%	100.0%	99.6%	100.0%	98.2%

**Table 5:** Pass rates calculated for the gamma evaluation in the tumor target and OARs compared with a reference TPS dose with the calculated dose in 3DVH and compass (C-Recal.: Compass Recalculation, C-Recon.: Compass Reconstruction) for the Prostate Plans.

		CTV	Pelvic LN	Bladder	Rectum	Small Bowel	Femoral Head	Colon
P1	3DVH	96.5%	99.5%	99.6%	99.9%		100.0%	100.0%
	C-Recal.	100.0%	100.0%	100.0%	100.0%		100.0%	100.0%
	C-Recon.	97.2%	99.9%	98.9%	100.0%		100.0%	100.0%
P2	3DVH	98.0%	99.9%	99.8%	99.6%	100.0%	100.0%	100.0%
	C-Recal.	100.0%	100.0%	100.0%	100.0%	100.0%	100.0%	100.0%
	C-Recon.	99.9%	100.0%	100.0%	100.0%	100.0%	100.0%	100.0%
P3	3DVH	98.4%	93.0%	99.9%	98.4%		100.0%	99.8%
	C-Recal.	100.0%	100.0%	100.0%	100.0%		100.0%	100.0%
	C-Recon.	94.5%	99.1%	98.4%	99.1%		100.0%	100.0%
P4	3DVH	95.7%	99.9%	99.5%	99.9%		100.0%	100.0%
	C-Recal.	100.0%	100.0%	100.0%	100.0%		100.0%	100.0%
	C-Recon.	98.4%	99.9%	99.7%	100.0%		100.0%	100.0%
P5	3DVH	98.3%	99.6%	99.9%	99.7%	100.0%	100.0%	99.9%
	C-Recal.	100.0%	100.0%	100.0%	100.0%	100.0%	100.0%	100.0%
	C-Recon.	98.3%	99.9%	99.5%	100.0%	100.0%	100.0%	100.0%
P6	3DVH	95.2%	99.9%	100.0%	99.8%	100.0%	100.0%	100.0%
	C-Recal.	100.0%	100.0%	100.0%	100.0%	100.0%	100.0%	100.0%
	C-Recon.	97.6%	100.0%	99.6%	100.0%	100.0%	100.0%	100.0%
P7	3DVH	97.9%	99.5%	98.4%	99.9%		100.0%	99.7%
	C-Recal.	91.5%	96.6%	99.7%	98.9%		100.0%	99.9%
	C-Recon.	47.2%	90.0%	83.2%	98.6%		100.0%	99.9%
P8	3DVH	95.5%	100.0%	99.3%	99.8%	100.0%	100.0%	99.9%
	C-Recal.	100.0%	100.0%	100.0%	100.0%	100.0%	100.0%	100.0%
	C-Recon.	97.1%	100.0%	98.8%	100.0%	100.0%	100.0%	100.0%
P9	3DVH	83.9%	99.1%	91.3%	99.7%	99.4%	100.0%	99.2%
	C-Recal.	89.8%	91.9%	99.7%	99.9%	100.0%	100.0%	99.9%
	C-Recon.	40.8%	83.2%	89.8%	99.9%	100.0%	100.0%	99.9%
P10	3DVH	92.9%	99.9%	99.9%	100.0%		100.0%	100.0%
	C-Recal.	100.0%	100.0%	100.0%	100.0%		99.8%	100.0%
	C-Recon.	98.5%	99.9%	99.6%	100.0%		100.0%	99.8%

**Table 6:** Differences in dose metrics of tumor target and OARs calculated using 3DVH and compass (C-Recal.: Compass Recalculation, C-Recon.: Compass Reconstruction) for the Head-Neck plans compared with a reference dose calculated using Eclipse TPS.

			CTV (D_90%_)	Brain- Stem (D_1%_)	Eyeball (D_mean_)	Lens (D_1%_)	Parotid (D_mean_)	Eso-phagus (D_1%_)	Cord (D_1%_)	Thyroid (D_mean_)	Hippo- campus (D_mean_)
H1	Reference Dose [cGy]		5011.8	3312.6	353.2	324.5					
	Difference [cGy]	3DVH	20.2	-43.6	-11.5	27.1					
		C-Recal.	-42.2	-78.3	-9.7	-9.5					
		C-Recon.	-9.5	-282.3	-15.5	-15.7					
H2	Reference Dose [cGy]		4752.4	3737.4			2203.4	5341.8	3703.7		
	Difference [cGy]	3DVH	-3.4	161.6			-5.1	1.2	44.3		
		C-Recal.	54.9	-5.4			9.1	50.8	-2.6		
		C-Recon.	84.7	-16.4			107.7	104.0	3.3		
H3	Reference Dose [cGy]		5325.4	180.0			722.1	4945.6	3805.7		
	Difference [cGy]	3DVH	22.4	0.0			-42.5	-92.6	48.5		
		C-Recal.	24.1	-17.9			15.2	36.8	7.5		
		C-Recon.	55	-5.8			40.7	226.6	41.0		
H4	Reference Dose [cGy]		4679.8						3137.9	2483.7	
	Difference [cGy]	3DVH	-9.8						104.9	81.7	
		C-Recal.	116.6						65.7	103.2	
		C-Recon.	158.5						25.3	374.1	
H5	Reference Dose [cGy]		5425.8	1912.2							548.0
	Difference [cGy]	3DVH	5.2	-25.6							-20.3
		C-Recal.	-40.7	-10.6							1.4
		C-Recon.	40.3	-8.0							-33.6
H6	Reference Dose [cGy]		3229.0	4757.6							
	Difference [cGy]	3DVH	8.0	98.4							
		C-Recal.	-14.6	-9.3							
		C-Recon.	135.0	148.9							
H7	Reference Dose [cGy]		3132.9	3094.0					3203.4		
	Difference [cGy]	3DVH	60.1	17.0					-1.4		
		C-Recal.	-7.5	-9.2					63.5		
		C-Recon.	46.7	-21.4					-6.8		
H8	Reference Dose [cGy]		5341.0	5321.1	645.3	369.8					
	Difference [cGy]	3DVH	-48.0	-16.1	-32.4	-17.7					
		C-Recal.	-76.3	-28.6	-14.4	-7.9					
		C-Recon.	37.9	25.0	-5.3	-45.9					
H9	Reference Dose [cGy]		5523.4	34.7			78.5	346.3	2971.0		
	Difference [cGy]	3DVH	8.6	-0.7			-3.4	-517.2	-13.0		
		C-Recal.	3.1	-3.3			-9.5	-21.2	-1.2		
		C-Recon.	47.1	3.3			-6.2	-537.7	-84.3		
H10	Reference Dose [cGy]		2562.7	2934.9	981.5	532.8					
	Difference [cGy]	3DVH	-7.7	12.1	2.8	16.7					
		C-Recal.	-25.9	-8.1	-23.9	-14.5					
		C-Recon.	4.2	36.5	54.2	41.6					

**Table 7:** Differences in dose metrics of tumor target and OARs calculated using 3DVH and compass (C-Recal.: Compass Recalculation, C-Recon.: Compass Reconstruction) for the Lung plans compared with a reference dose calculated using Eclipse TPS.

			CTV(D_90%_)	Lung (D_mean_)	Lung (D_1%_)	Heart (D_mean_)	Heart (D_1%_)	Cord (D_1%_)
L1	Reference Dose [cGy]		3106.5	1899.7	4373.4	589.5	2482.9	2567.2
	Difference [cGy]	3DVH	-20.5	-1.7	28.6	-19.2	-2.9	-31.2
		C-Recal.	41.7	-2.7	131.8	6.9	56.6	47.6
		C-Recon.	44.9	8.7	161.3	-4.9	56.9	49.4
L2	Reference Dose [cGy]		4912.4	135.2	2122.2	7.6	16.4	399.7
	Difference [cGy]	3DVH	65.6	3.3	70.8	-6.84	-0.4	-32.7
		C-Recal.	43.5	-4.6	49.5	-7.1	-11.5	23.1
		C-Recon.	131.0	0.0	36.0	12.8	22.0	21.3
L3	Reference Dose [cGy]		4924.1	592.9	5579.5	27.3	57.6	2708.4
	Difference [cGy]	3DVH	-19.1	-10.2	15.5	0.0	-0.6	-129.4
		C-Recal.	13.6	-21.1	-9.2	-15.7	-16.4	41.3
		C-Recon.	0.2	-14.3	-13.2	20.8	19.7	1.4
L4	Reference Dose [cGy]		3850.7	1347.9	5408.0	1585.8	5436.0	4455.7
	Difference [cGy]	3DVH	35.3	-5.4	59	-40.7	15.0	-43.7
		C-Recal.	32.7	-23.1	118.3	29.1	94.4	96.9
		C-Recon.	49.4	-15.4	142.8	4.0	134.2	55.0
L5	Reference Dose [cGy]		4481.7	1314.3	6014.8	252.8	663.8	4433.0
	Difference [cGy]	3DVH	-3.7	-7.3	69.2	-8.8	-0.2	-5.0
		C-Recal.	63.1	-18.2	158.9	-14.6	2.6	95.8
		C-Recon.	46.2	-13.9	194.0	-1.8	-59.2	128.7
L6	Reference Dose [cGy]		4732.5	902.5	5049.8	202.7	1488.14	4233.8
	Difference [cGy]	3DVH	75.5	-6.9	84.2	-12.6	-51.1	-25.8
		C-Recal.	8.1	-23.7	35.6	-23.7	24.7	131.2
		C-Recon.	23.1	-12.4	85.1	-2.0	-102.3	153.6
L7	Reference Dose [cGy]		4929.8	243.9	3040.6	16.2	42.9	446.3
	Difference [cGy]	3DVH	-37.8	0.3	-15.6	0.4	-5.9	-65.3
		C-Recal.	19.7	-4.1	19.0	-7.1	-9.9	29.4
		C-Recon.	99.0	-5.8	5.4	4.2	-7.3	74.3
L8	Reference Dose [cGy]		4870.1	987.4	6325.5	190.9	1243.8	2166.4
	Difference [cGy]	3DVH	6.9	-14.1	69.5	-17.0	-128.8	-0.4
		C-Recal.	98.7	10.9	90.2	-0.5	6.0	6.6
		C-Recon.	25.1	-13.5	153.1	-26.9	-234.1	-20.0
L9	Reference Dose [cGy]		4445.2	1246.0	5347.6	864.0	4405.8	4453.5
	Difference [cGy]	3DVH	74.8	4.0	78.4	1.2	75.2	-34.5
		C-Recal.	93.8	-48.5	171.6	-4.0	94.4	99.2
		C-Recon.	10.3	-42.9	47.0	-6.7	23.4	-128.4
L10	Reference Dose [cGy]		4306.2	1214.1	5024.9	1931.3	4733.3	4307.5
	Difference [cGy]	3DVH	-20.2	1.8	13.1	14.0	34.7	-70.5
		C-Recal.	21.5	-53.1	0.2	16.6	48.0	91.7
		C-Recon.	83.3	-1.0	71.7	59.9	118.4	119.1

**Table 8:** Differences in dose metrics of tumor target and OARs calculated using 3DVH and compass (C-Recal.: Compass Recalculation, C-Recon.: Compass Reconstruction) for the Prostate plans compared with a reference dose calculated using Eclipse TPS.

			CTV(D_90%_)	Pelvic LN (D_90%_)	Rectum (D_mean_)	Rectum (D_1%_)	Bladder (D_mean_)	Bladder (D_1%_)
P1	Reference Dose [cGy]		5232.6	4524.9	2973.0	5252.2	3551.1	5259.1
	Difference [cGy]	3DVH	96.4	-40.9	-17.0	54.8	-21.1	69.9
		C-Recal.	47.6	-12.9	-29.4	78.7	-26.4	17.9
		C-Recon.	124.9	-23.5	-111.4	-40.9	74.2	227.4
P2	Reference Dose [cGy]		5373.9	4538.6	2916.6	5259.3	3640.7	5280.5
	Difference [cGy]	3DVH	93.1	-74.6	8.4	67.7	-23.7	68.5
		C-Recal.	-22.0	61.9	-62.9	20.2	-67.7	-42.4
		C-Recon.	57.5	-74.6	-153.4	-76.6	58.4	167.4
P3	Reference Dose [cGy]		4425.2	4437.7	2331.3	4475.9	3165.6	4507.7
	Difference [cGy]	3DVH	27.8	-99.7	-58.3	29.1	-3.6	20.3
		C-Recal.	13.8	-28.9	-24.7	69.1	-34.9	18.2
		C-Recon.	83.8	-39.3	-93.4	39.8	55.4	150.5
P4	Reference Dose [cGy]		4450.8	4440.4	2520.7	4486.1	3066.6	4481.2
	Difference [cGy]	3DVH	70.2	-24.4	6.3	79.9	9.4	66.8
		C-Recal.	-1.1	-39.5	-41.0	36.8	-49.8	-23.3
		C-Recon.	73.5	-40.7	-150.1	-45.3	30.0	120.1
P5	Reference Dose [cGy]		5168.8	4545.2	2828.7	5267.8	3550.7	5225.4
	Difference [cGy]	3DVH	-31.6	-65.2	9.3	29.2	-37.7	-13.4
		C-Recal.	20.5	-29.2	-45.0	33.2	-41.2	20.6
		C-Recon.	107.1	-38.5	-126.8	-70.8	36.3	168.3
P6	Reference Dose [cGy]		5198.7	4518.3	3066.1	5330.6	3582.9	5287.0
	Difference [cGy]	3DVH	65.3	-43.3	25.9	115.4	-13.9	12.7
		C-Recal.	13.3	-26.7	-30.6	75.1	-42.2	6.8
		C-Recon.	119.4	-37.1	-160.3	8.1	99.4	159.2
P7	Reference Dose [cGy]		4449.5	4436.2	2507.9	4475.8	3278.6	4508.7
	Difference [cGy]	3DVH	68.5	-32.2	-31.9	51.2	12.4	103.3
		C-Recal.	116.7	89.9	21.2	165.6	32.3	115.6
		C-Recon.	480.0	83.3	-29.6	168.5	175.2	235.9
P8	Reference Dose [cGy]		5376.3	4593.6	2947.6	5268.6	3649.7	5301.4
	Difference [cGy]	3DVH	183.7	-47.6	-47.6	75.4	-46.7	80.6
		C-Recal.	29.0	-18.8	-33.7	55.1	-37.6	9.7
		C-Recon.	72.4	-46.6	-163.2	-167.3	145.0	251.8
P9	Reference Dose [cGy]		4428.4	4477.6	2291.7	4482.2	3155.4	4507.2
	Difference [cGy]	3DVH	220.6	0.4	15.3	156.8	90.6	263.8
		C-Recal.	99.9	100.8	13.5	203.7	43.9	112.0
		C-Recon.	212.8	110.4	-46.5	132.8	142.8	269.3
P10	Reference Dose [cGy]		5399.6	4525.4	2917.8	5265.0	3456.3	5243.7
	Difference [cGy]	3DVH	114.4	-45.4	8.2	113	-45.3	99.3
		C-Recal.	17.6	-31.0	-26.4	68.0	-42.9	24.5
		C-Recon.	66.8	-31.5	-121.9	-17.5	85.5	240.5

**Fig 7:**
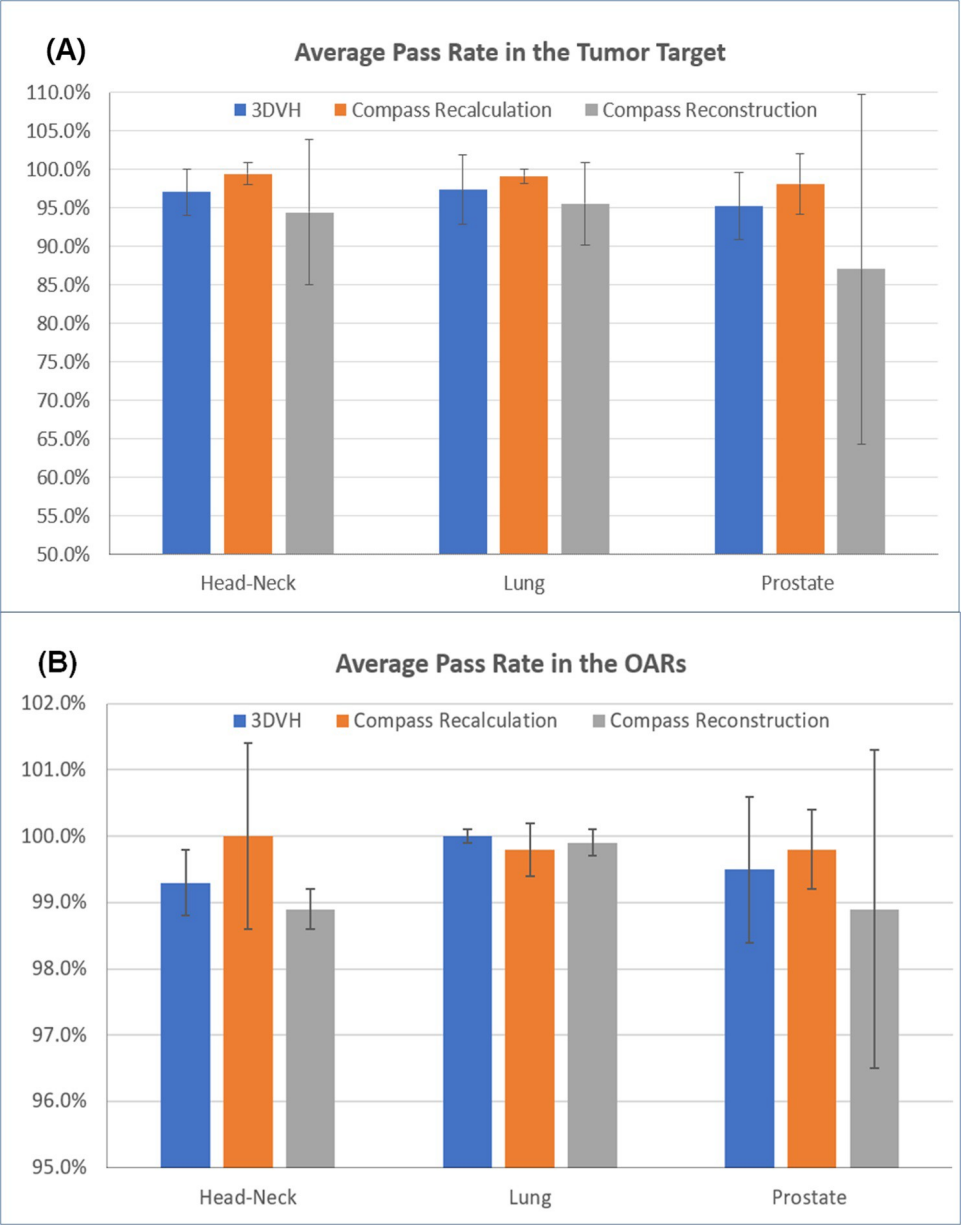
Comparison of the pass rate calculated in a gamma evaluation between a reference dose calculated using Eclipse TPS and a calculated dose using 3DVH and compass. (A) average pass rate in the tumor target, (B) average pass rate in the OARs.

Fig 7 shows the average pass rates in the tumor target and OARs according to the calculation method for each treatment site. The average pass rates in the tumor target according to the dose calculation methods were calculated as 96.5±3.9% in 3DVH, 98.9±2.1% in Compass recalculation, and 92.3±12.5% in Compass reconstruction. The average pass rates in OARs according to the dose calculation methods were calculated as 99.6±0.6% in 3DVH, 99.9±0.3% in Compass recalculation and 99.2±1.0% in Compass reconstruction.

**Fig 8:**
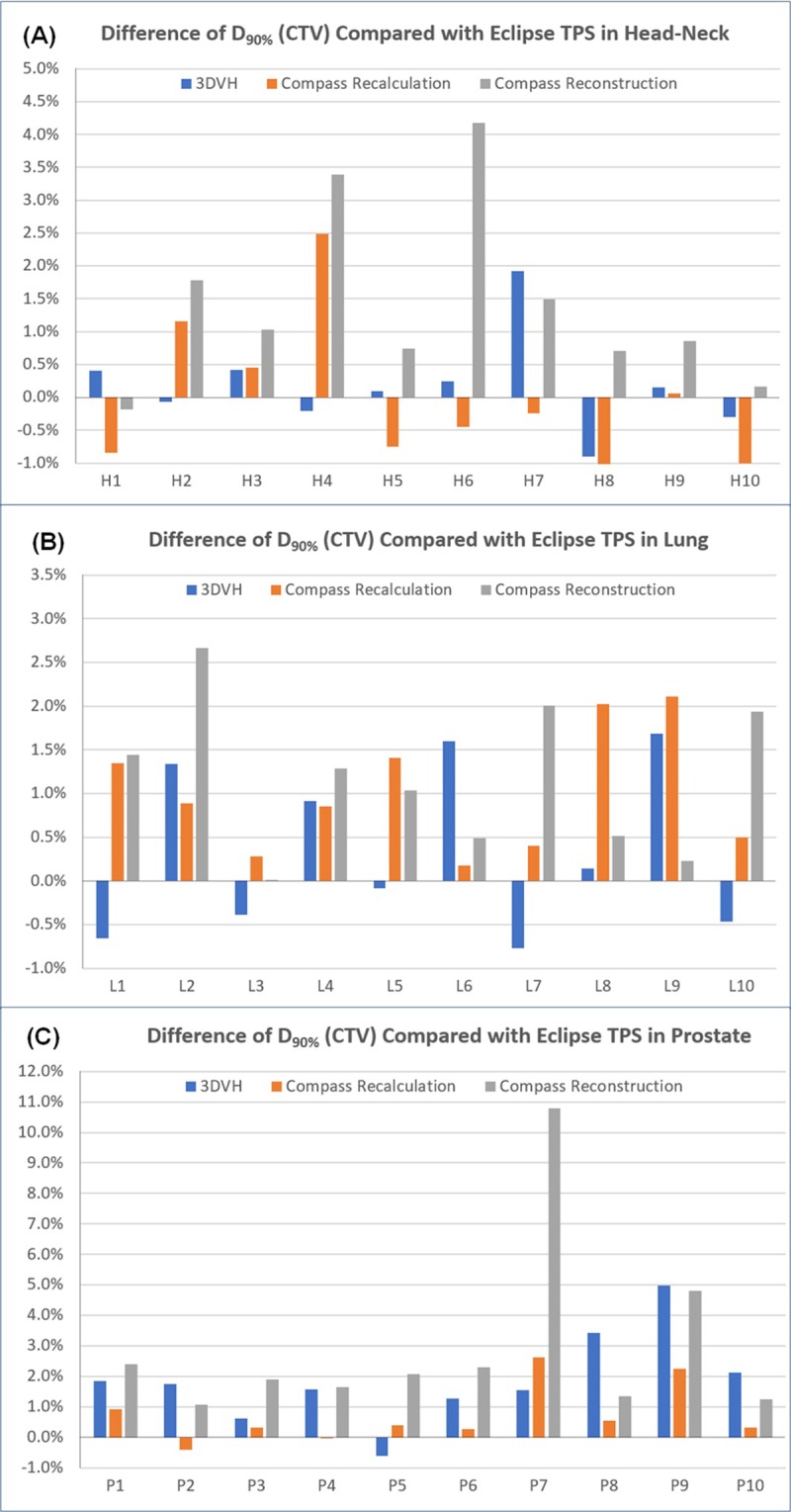
Difference of D_90%_ (CTV) calculated using 3DVH and compass compared with a reference value calculated in Eclipse TPS. (A) head-neck plans, (B) lung plans, (C) prostate plans.

Tables 6–8 show the variation in the major dose metrics of the tumor target and OARs according to the three dose calculation methods. Compared with the change in the average pass rate in the gamma evaluation, the changes in the dose metric values occurred more variously according to the dose calculation methods. Fig 8 shows the difference of a calculated D_90%_ (CTV) using 3DVH and Compass compared with a reference value calculated in Eclipse TPS. The average differences of D_90%_ (CTV) were 0.78±1.32% in 3DVH, 0.55±1.04% in Compass recalculation and 1.84±2.04% in Compass reconstruction. The average differences of calculated dose metrics in OARs using 3DVH and Compass compared with reference values calculated in Eclipse TPS were -1.22±8.81% in 3DVH, -2.61±13.11% in Compass recalculation and 2.58±15.36% in Compass reconstruction. As can be seen from these results, there were significant variations in the differences of main dose metrics in OARs compared with those of D_90%_ (CTV). This showed that more attention should be paid to the analysis on the dose metrics of OARs calculated in the patient’s dose reconstruction QA tool.

In both CTV and OARs, the differences between the results from Compass reconstruction calculation and the reference value were significantly larger (*p* < 0.03) than those of 3DVH and Compass recalculation. This was due to the characteristics of calculating the dose based on the measurement data that was influenced by the factors which could occur during the measurement process such as the variation of the output and mechanical accuracy in the linear accelerator, and the sensitivity variation of the measurement device.

## Discussion

In this study, the dosimetric difference compared with the actual dose data measured using ArcCHECK was analyzed in order to evaluate the accuracy of dose calculation in Compass to develop a dose reconstruction program for IMRT DQA. Based on the results of gamma evaluation for 12 plans, Compass achieved an average pass rate of more than 98% in both recalculation and reconstruction methods. This showed a dosimetric accuracy similar to that of Eclipse TPS, which achieved a pass rate of 98.7%. Thus, the dosimetric accuracy related to the beam commissioning process during the installation of Compass can be confirmed, and the reliability of dose results can be guaranteed when calculating the dose distribution in a patient’s body before applying it to clinical cases.

Although many studies have demonstrated the accuracy of a commercially available dose reconstruction program for IMRT DQA [11,19–23], it is reasonable that the accuracy of the dose calculated using the program should be verified in advance through a comparison with the actual measured dose distribution before application in practical clinical cases. Accordingly, the verification of dosimetric accuracy based on the dose measured using the detector array for IMRT DQA, as devised in this study, can be effectively used in clinical sites.

Comparing the dose calculation results obtained using Compass and 3DVH, the pass rate in gamma evaluation was slightly different according to the characteristics of the calculation program and the positions and shapes of the tumor target and OARs.

In the tumor target, the average pass rate was 98.9±2.1% in the Compass recalculation, 96.5±3.9% in 3DVH, and 92.3±12.5% in the Compass reconstruction. In the OARs, the average pass rate was 99.9±0.3% in the Compass recalculation, 99.6±0.6% in 3DVH, and 99.2±1.0% in the Compass reconstruction, demonstrating no significant difference for the different calculation methods in this case. The reason for the differences in the pass rate results in cases where the tumor target is larger is because the dose difference calculated according to the program algorithm is relatively larger because the target region is irradiated with a larger amount of dose. The reason for the lowest pass rate in the Compass reconstruction is the occurrence of various possible errors in the measurement process, which could be included in the calculation of the dose based on the MatriXX measurement data. The pass rates in the tumor target and OARs showed acceptable values, guaranteeing a certain level of accuracy, except for a few cases as shown in Tables 3–5.

Comparisons of important dose metric values on DVH that are related to actual tumor control and complication probability are also needed rather than a simple comparison of gamma evaluation values in CTV and OARs. As shown in Tables 6–8, the calculation results of important dose metrics for tumor target and OARs showed various differences according to the program calculation method and the position of the target and OARs. This confirmed that a detailed analysis of the important dose metrics related to tumor control and complications of OARs should be performed additionally rather than merely relying on the gamma evaluation results when analyzing the characteristics of the dose distribution calculated using the dose reconstruction program. And an appropriate tolerance range in the dose metric analysis should be set up for the proper use of patient dose QA tool such as compass.

The additional study on the dose characteristics calculated by compass according to the various cases will be performed when the sufficient compass dosimeric results are collected for more patient cases.

## Conclusions

In this study, we designed and verified the validity of a method that utilizes the actual dose measured using ArcCHECK, the IMRT DQA detector array, to validate the dose accuracy calculated using Compass, the dose reconstruction program, inside a patient’s body. Before the commercialized dose reconstruction program is used at a clinical site, it is necessary to verify the accuracy of the dose calculated using the program. Using the method developed in this study, the dose accuracy is expected to be determined efficiently by comparing it with the actual measured dose distribution.

## Supporting information

S1 Fig 6MV-RapidArc Dose Recalculated by Compass for Head-Neck Case. (JPG)

S2 Fig 6MV-RapidArc Dose Reconstructed by Compass for Head-Neck Case. (JPG)

S3 Fig 6MV-IMRT Dose Recalculated by Compass for Head-Neck Case. (JPG)

S4 Fig 6MV-IMRT Dose Reconstructed by Compass for Head-Neck Case. (JPG)

S5 Fig 10MV-RapidArc Dose Recalculated by Compass for Head-Neck Case. (JPG)

S6 Fig 10MV-RapidArc Dose Reconstructed by Compass for Head-Neck Case. (JPG)

S7 Fig 10MV-IMRT Dose Recalculated by Compass for Head-Neck Case. (JPG)

S8 Fig 10MV-IMRT Dose Reconstructed by Compass for Head-Neck Case. (JPG)

S9 Fig 6MV-RapidArc Dose Recalculated by Compass for Lung Case. (JPG)

S10 Fig 6MV-RapidArc Dose Reconstructed by Compass for Lung Case. (JPG)

S11 Fig 6MV-IMRT Dose Recalculated by Compass for Lung Case. (JPG)

S12 Fig 6MV-IMRT Dose Reconstructed by Compass for Lung Case. (JPG)

S13 Fig 10MV-RapidArc Dose Recalculated by Compass for Lung Case. (JPG)

S14 Fig 10MV-RapidArc Dose Reconstructed by Compass for Lung Case. (JPG)

S15 Fig 10MV-IMRT Dose Recalculated by Compass for Lung Case. (JPG)

S16 Fig 10MV-IMRT Dose Reconstructed by Compass for Lung Case. (JPG)

S17 Fig 6MV-RapidArc Dose Recalculated by Compass for Prostate Case. (JPG)

S18 Fig 6MV-RapidArc Dose Reconstructed by Compass for Prostate Case. (JPG)

S19 Fig 6MV-IMRT Dose Recalculated by Compass for Prostate Case. (JPG)

S20 Fig 6MV-IMRT Dose Reconstructed by Compass for Prostate Case. (JPG)

S21 Fig 10MV-RapidArc Dose Recalculated by Compass for Prostate Case. (JPG)

S22 Fig 10MV-RapidArc Dose Reconstructed by Compass for Prostate Case. (JPG)

S23 Fig 10MV-IMRT Dose Recalculated by Compass for Prostate Case. (JPG)

S24 Fig 10MV-IMRT Dose Reconstructed by Compass for Prostate Case. (JPG)
