# Supplementary figures and images for: Dosimetric evaluation of the compass program for patient dose analysis in IMRT delivery quality assurance
Source: PLoS One. 2018 Dec 20;13(12):e0209180. doi: 10.1371/journal.pone.0209180 (PMC6301628; doi:10.1371/journal.pone.0209180)

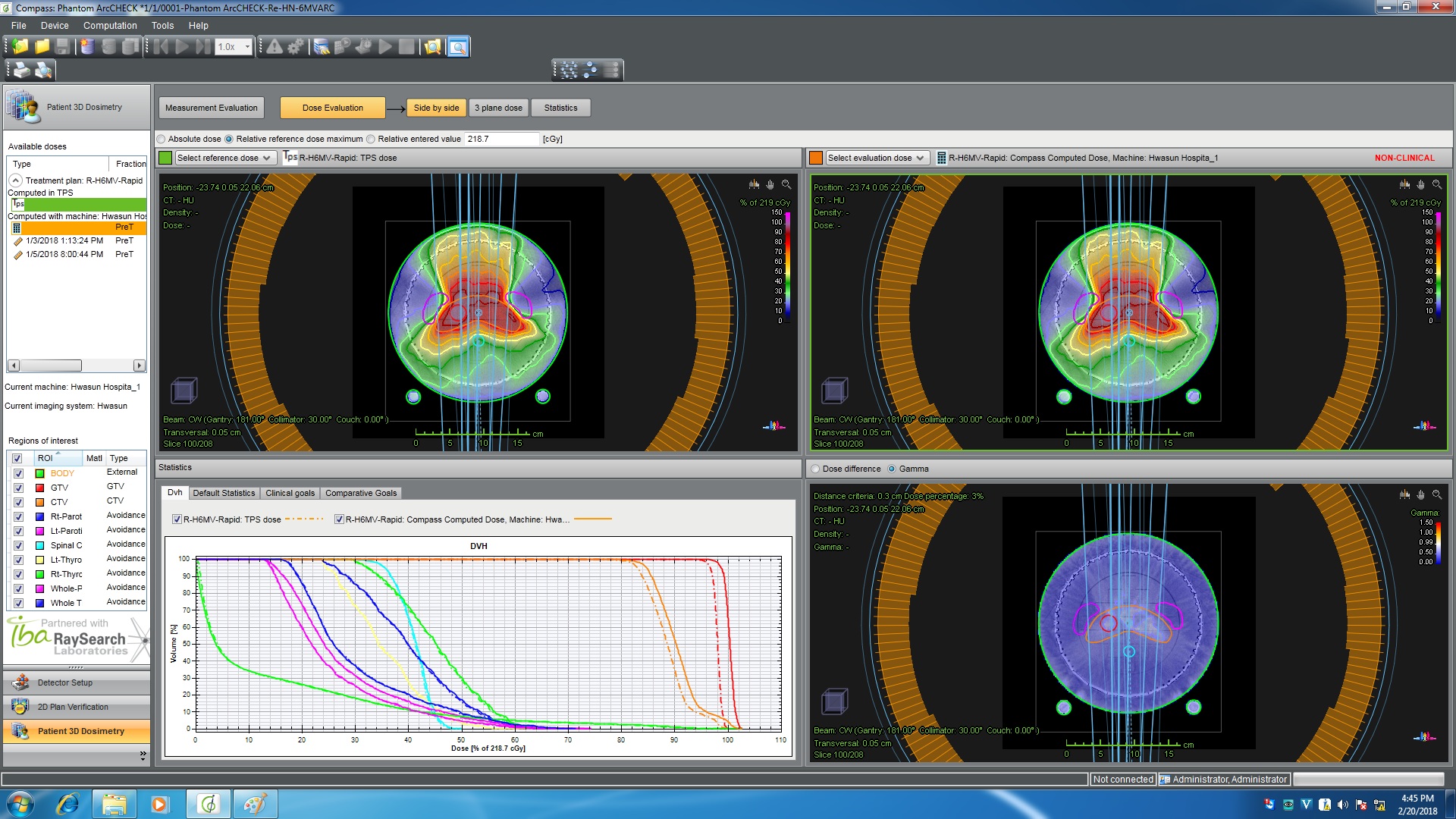

Supplement: S1 Fig — (JPG) [file pone.0209180.s001.jpg]

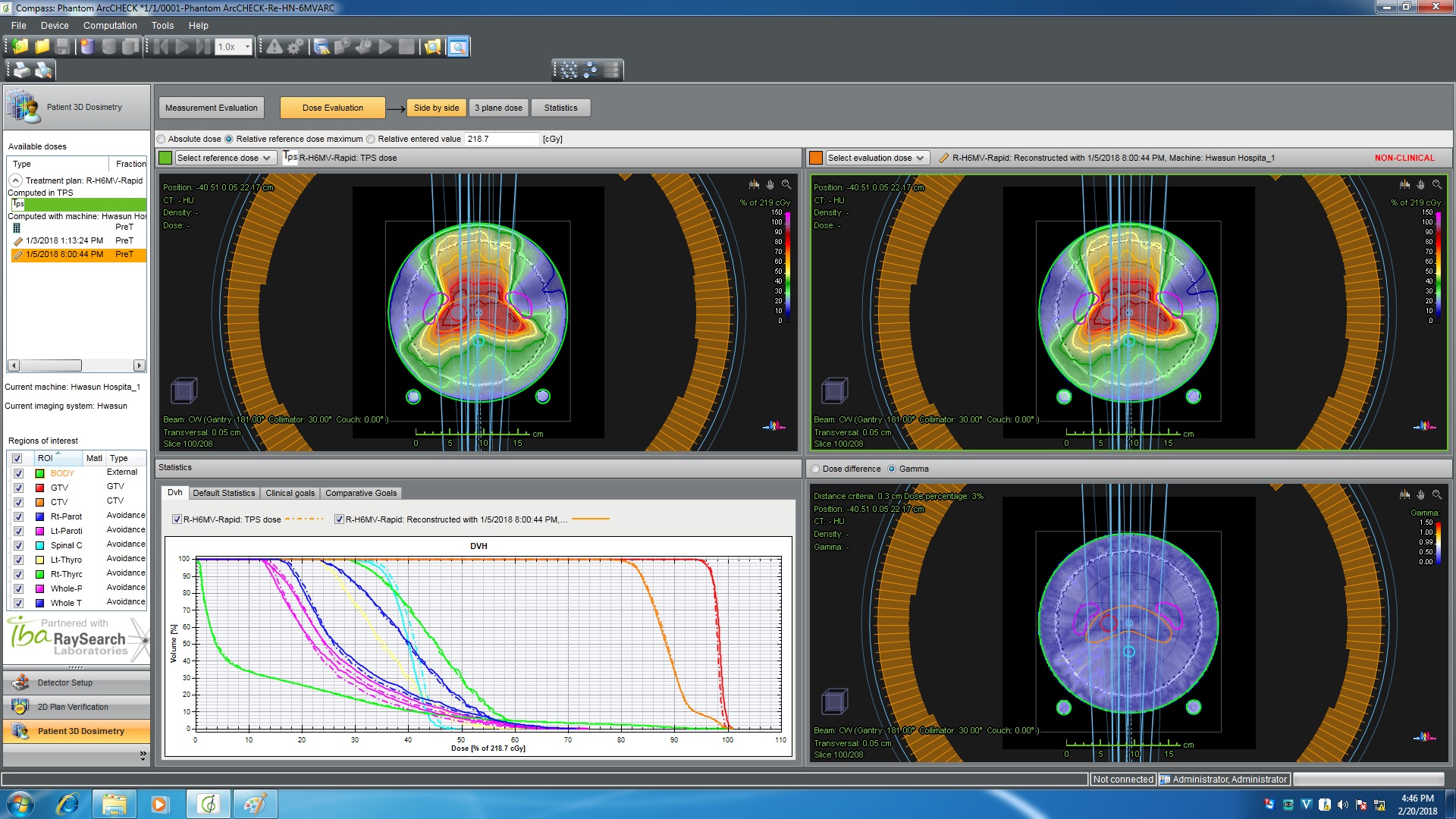

Supplement: S2 Fig — (JPG) [file pone.0209180.s002.jpg]

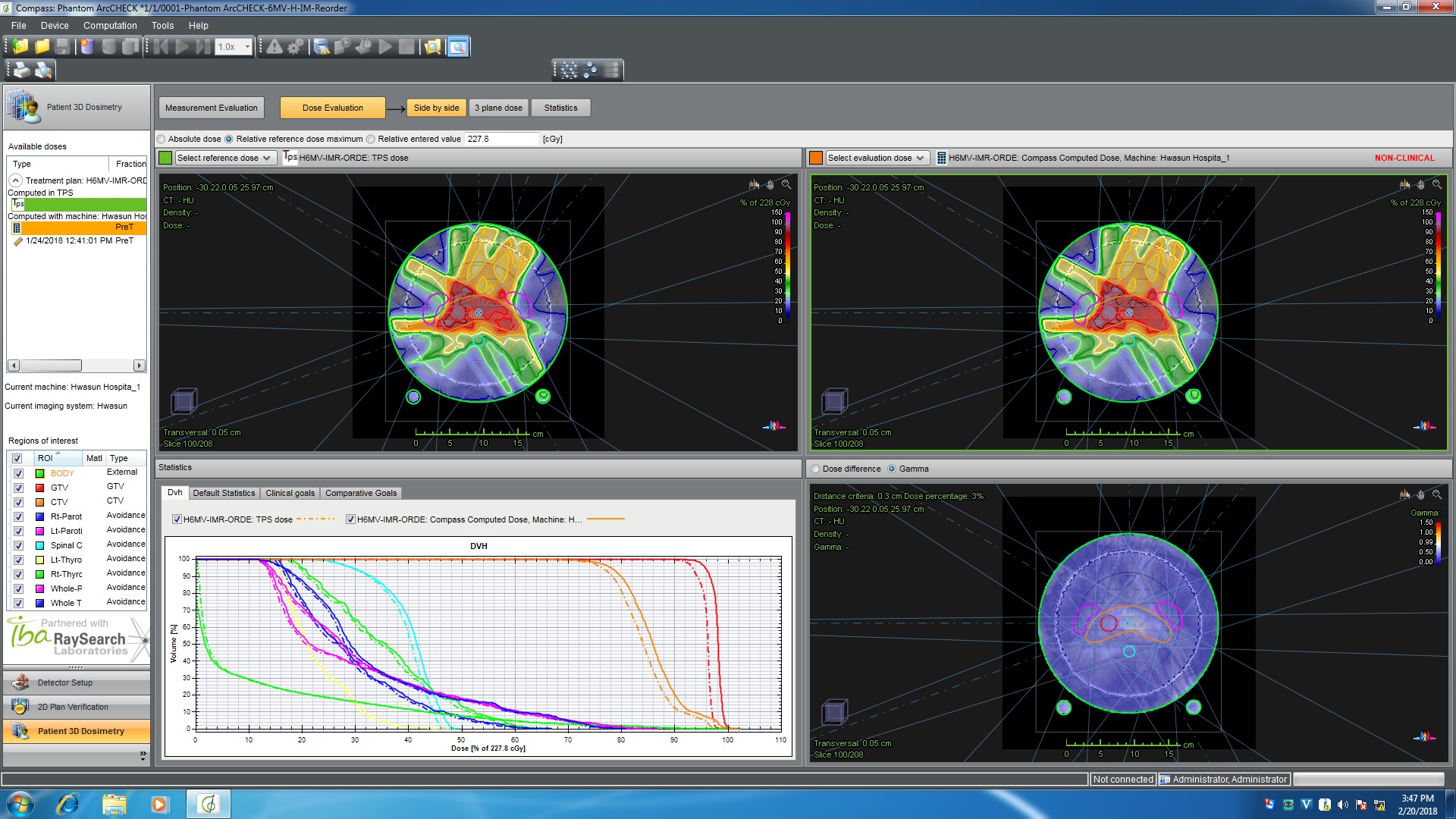

Supplement: S3 Fig — (JPG) [file pone.0209180.s003.jpg]

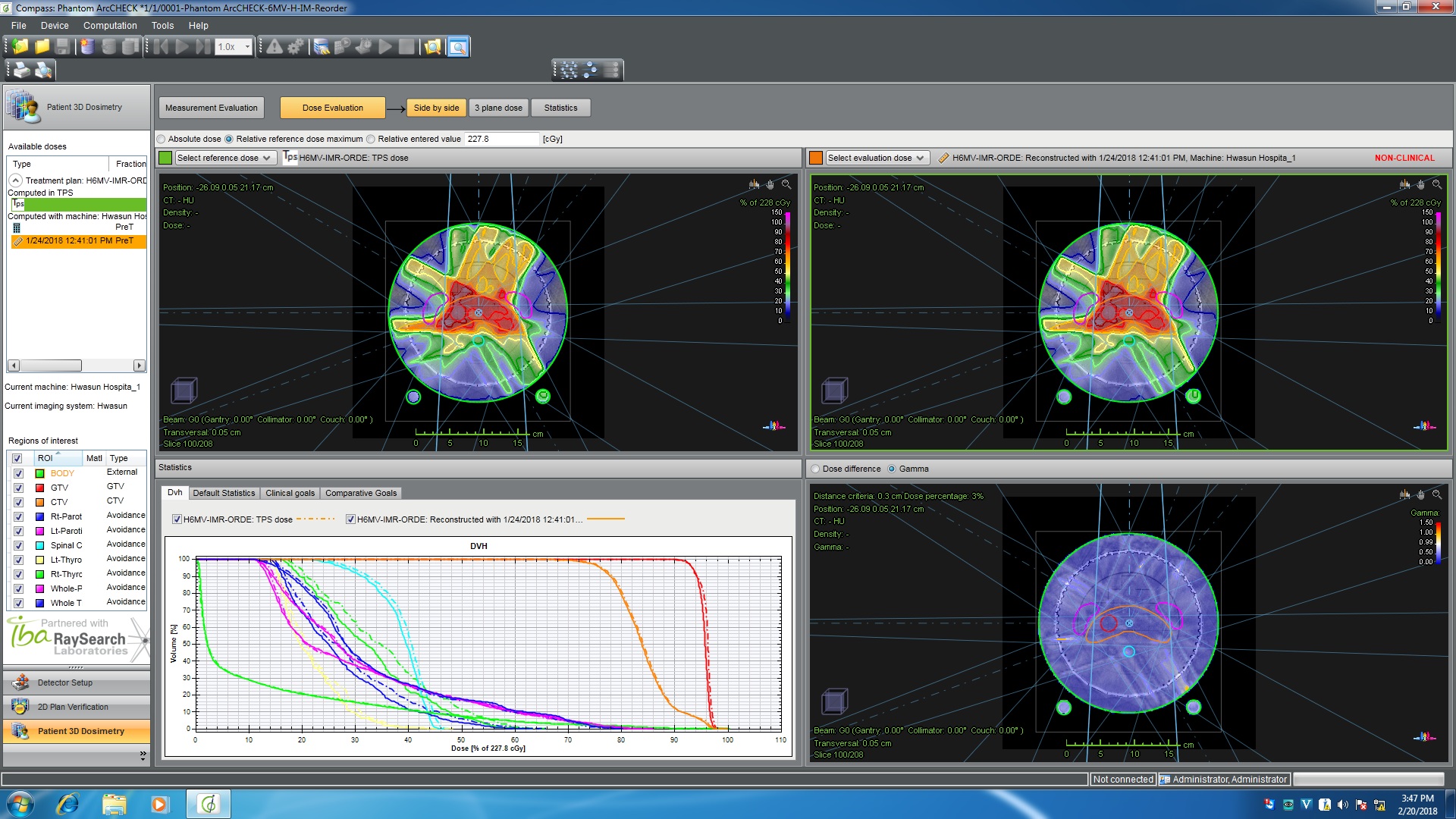

Supplement: S4 Fig — (JPG) [file pone.0209180.s004.jpg]

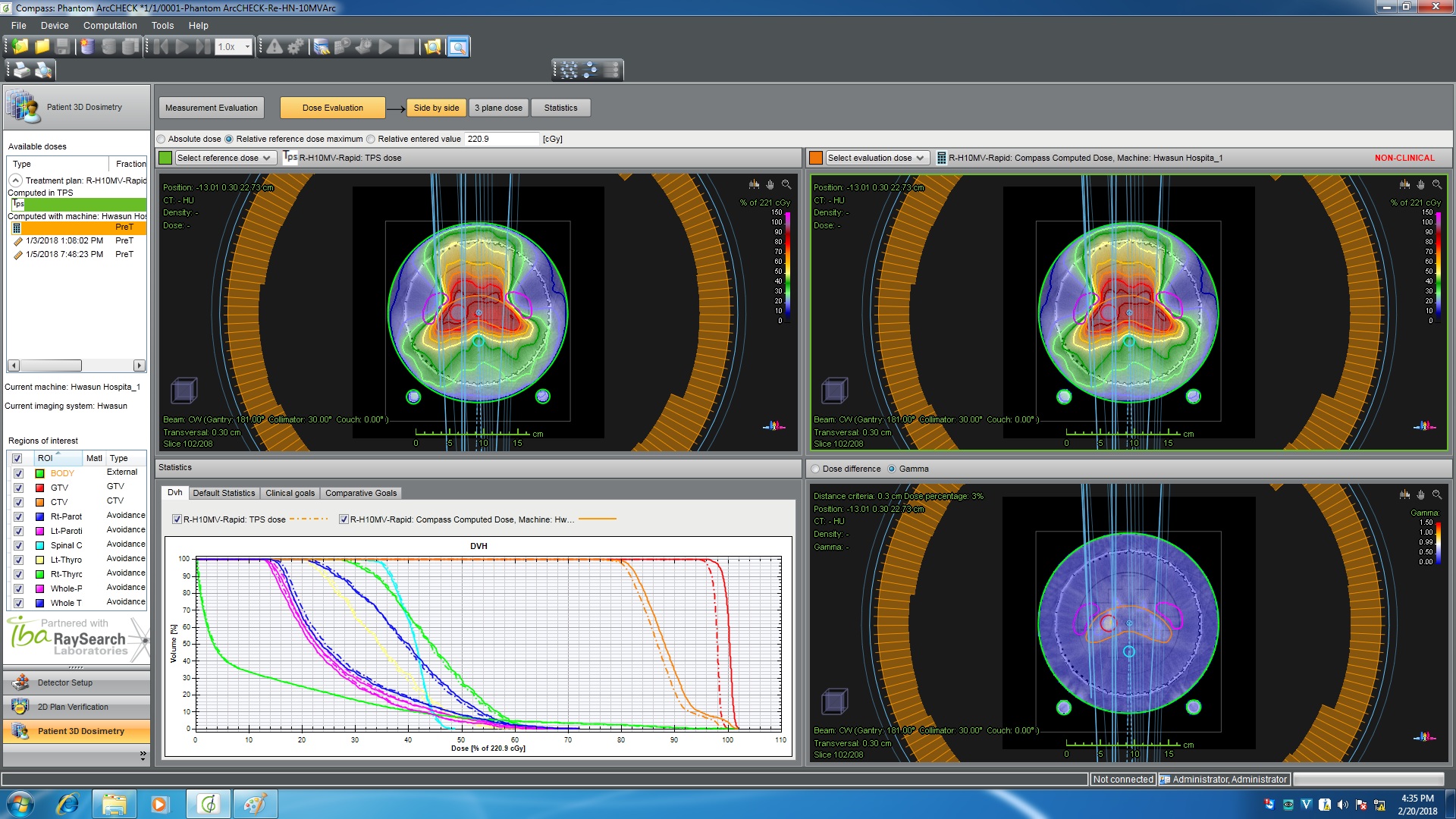

Supplement: S5 Fig — (JPG) [file pone.0209180.s005.jpg]

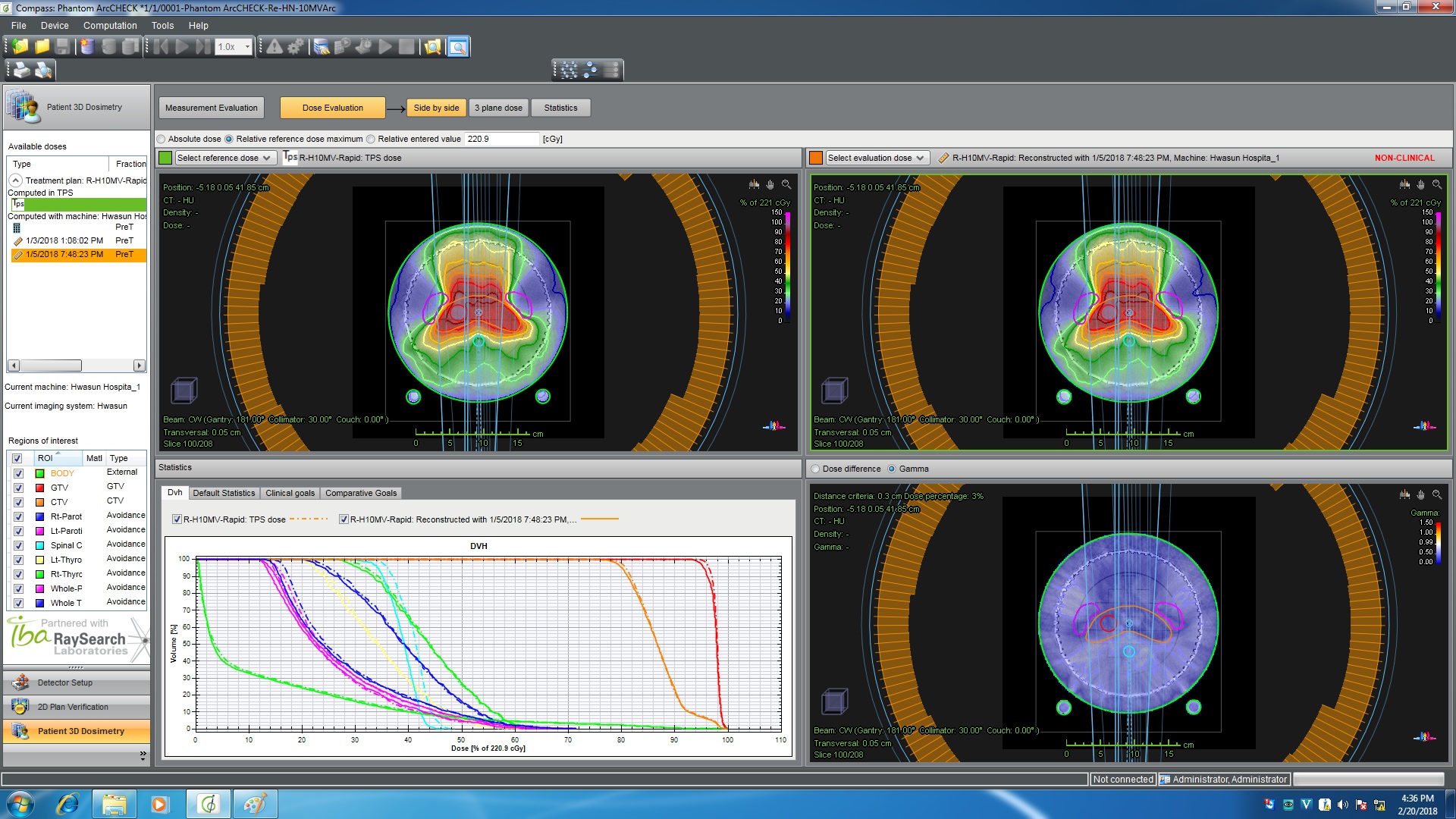

Supplement: S6 Fig — (JPG) [file pone.0209180.s006.jpg]

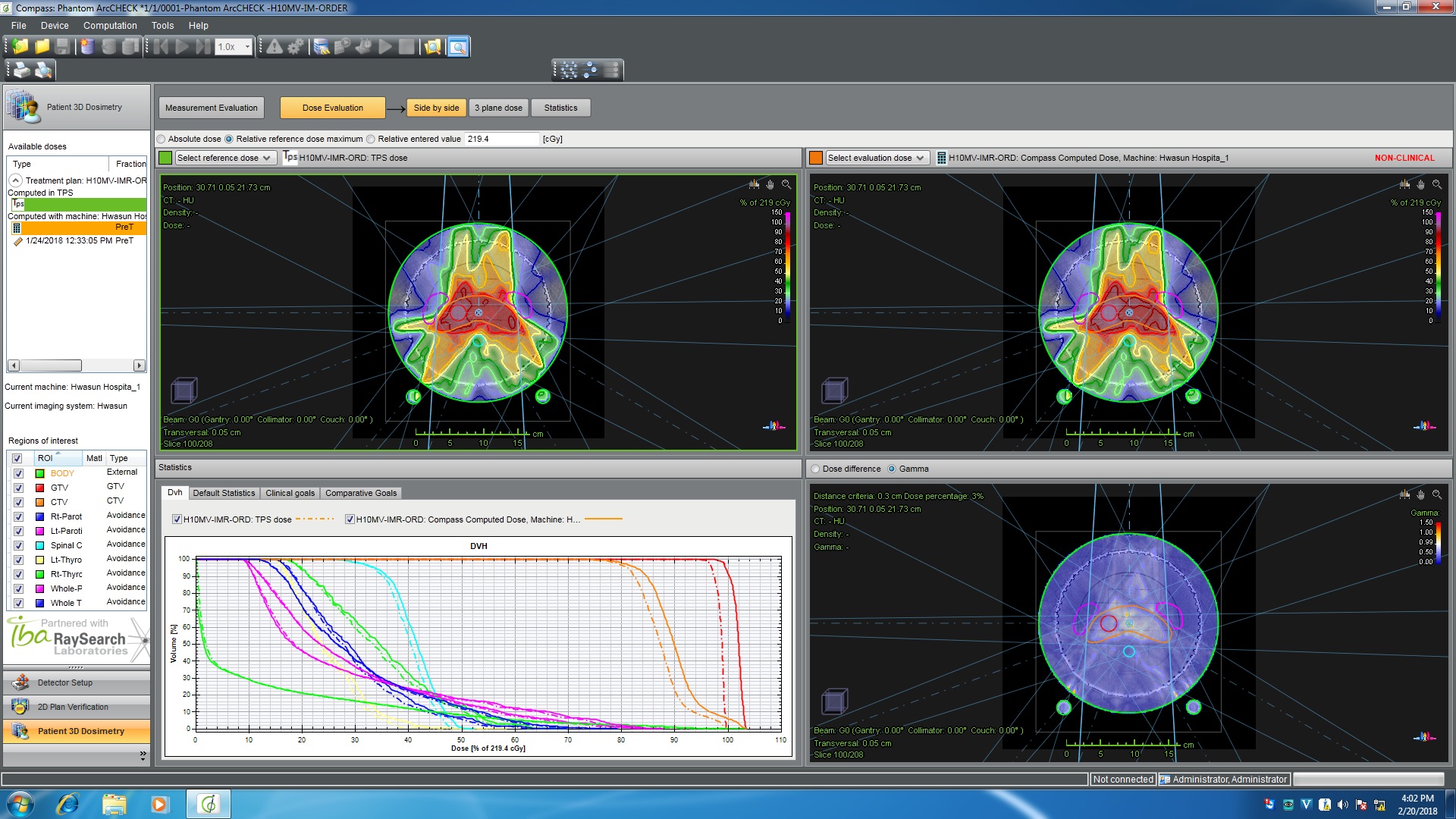

Supplement: S7 Fig — (JPG) [file pone.0209180.s007.jpg]

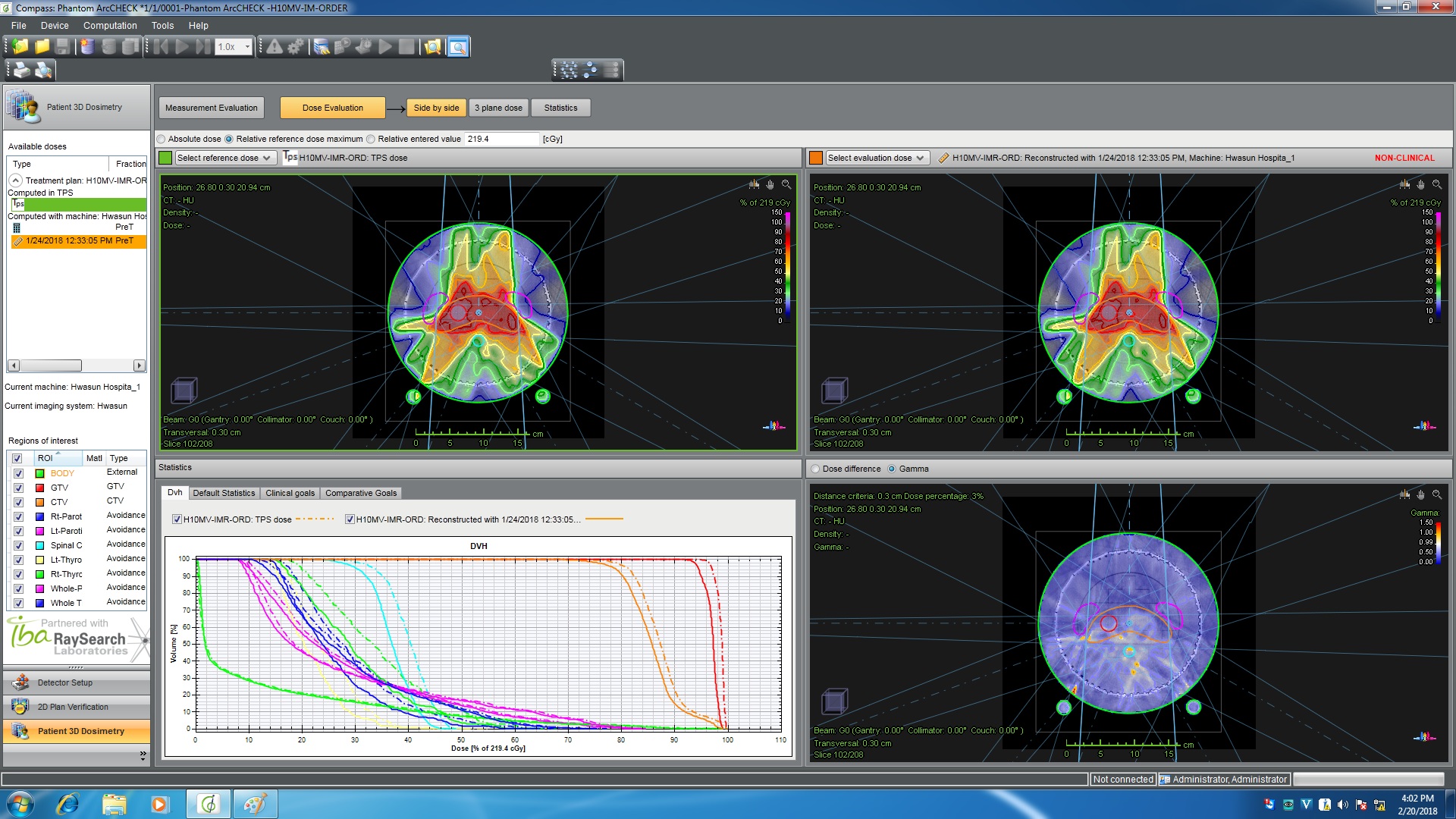

Supplement: S8 Fig — (JPG) [file pone.0209180.s008.jpg]

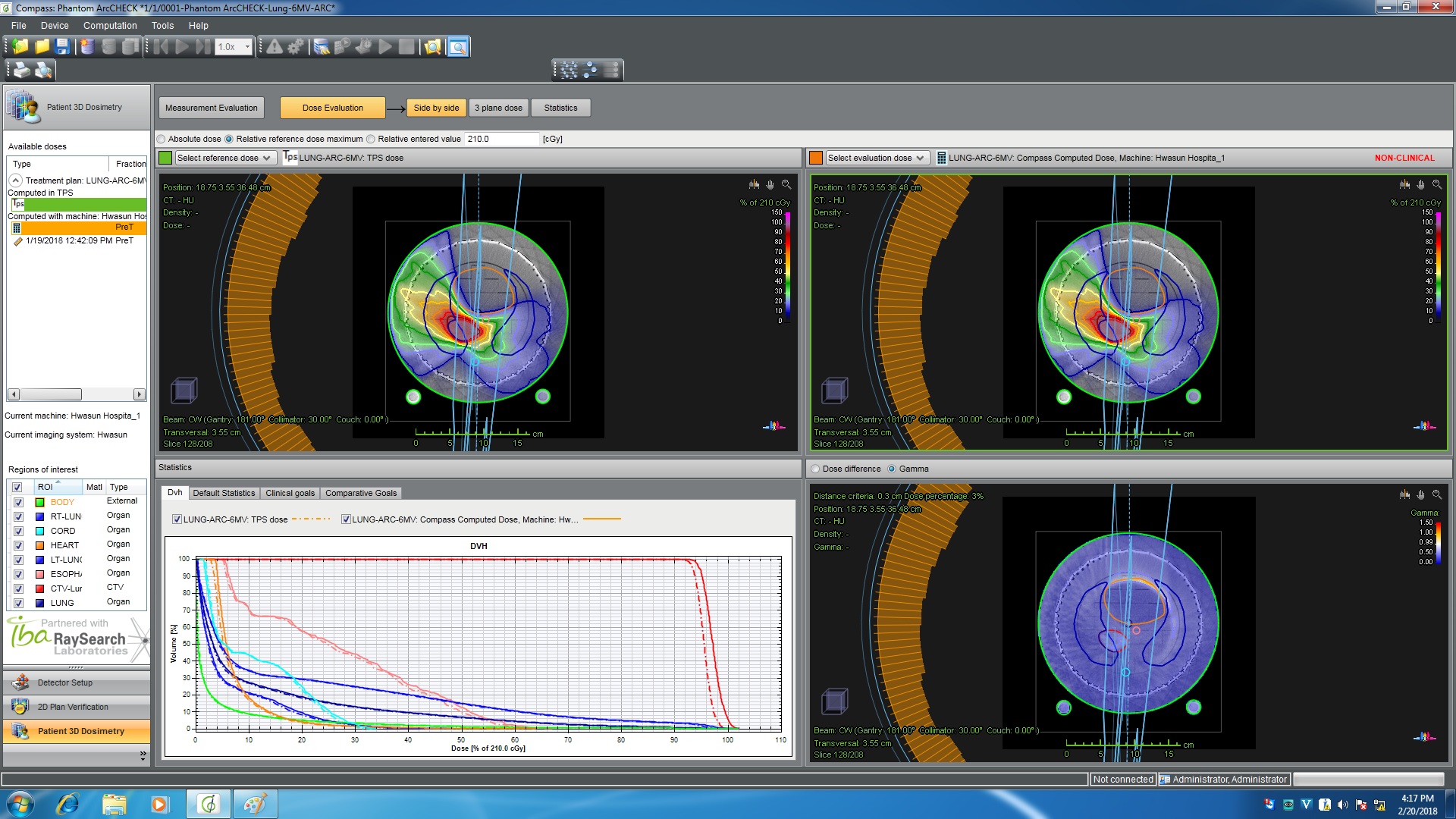

Supplement: S9 Fig — (JPG) [file pone.0209180.s009.jpg]

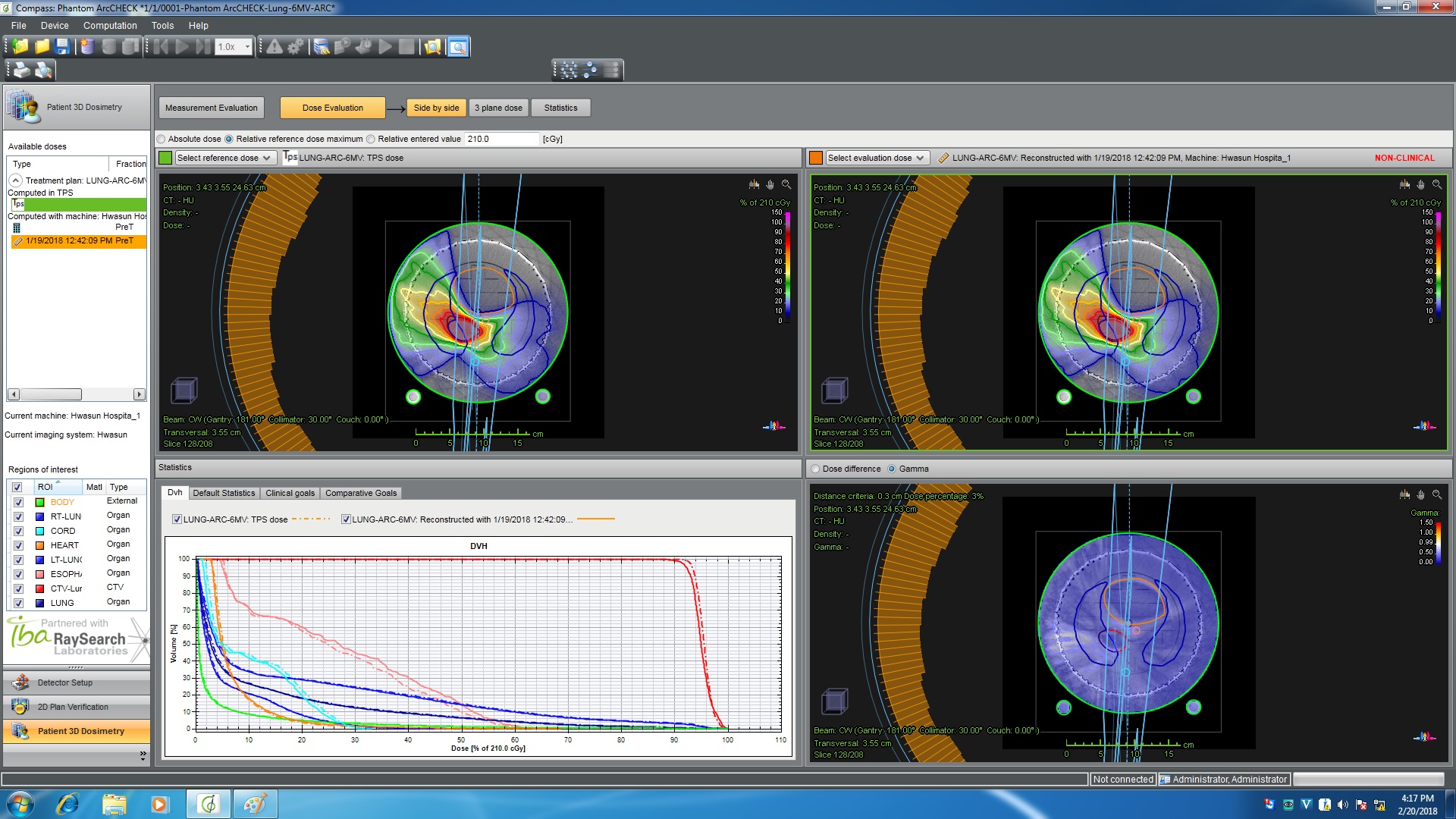

Supplement: S10 Fig — (JPG) [file pone.0209180.s010.jpg]

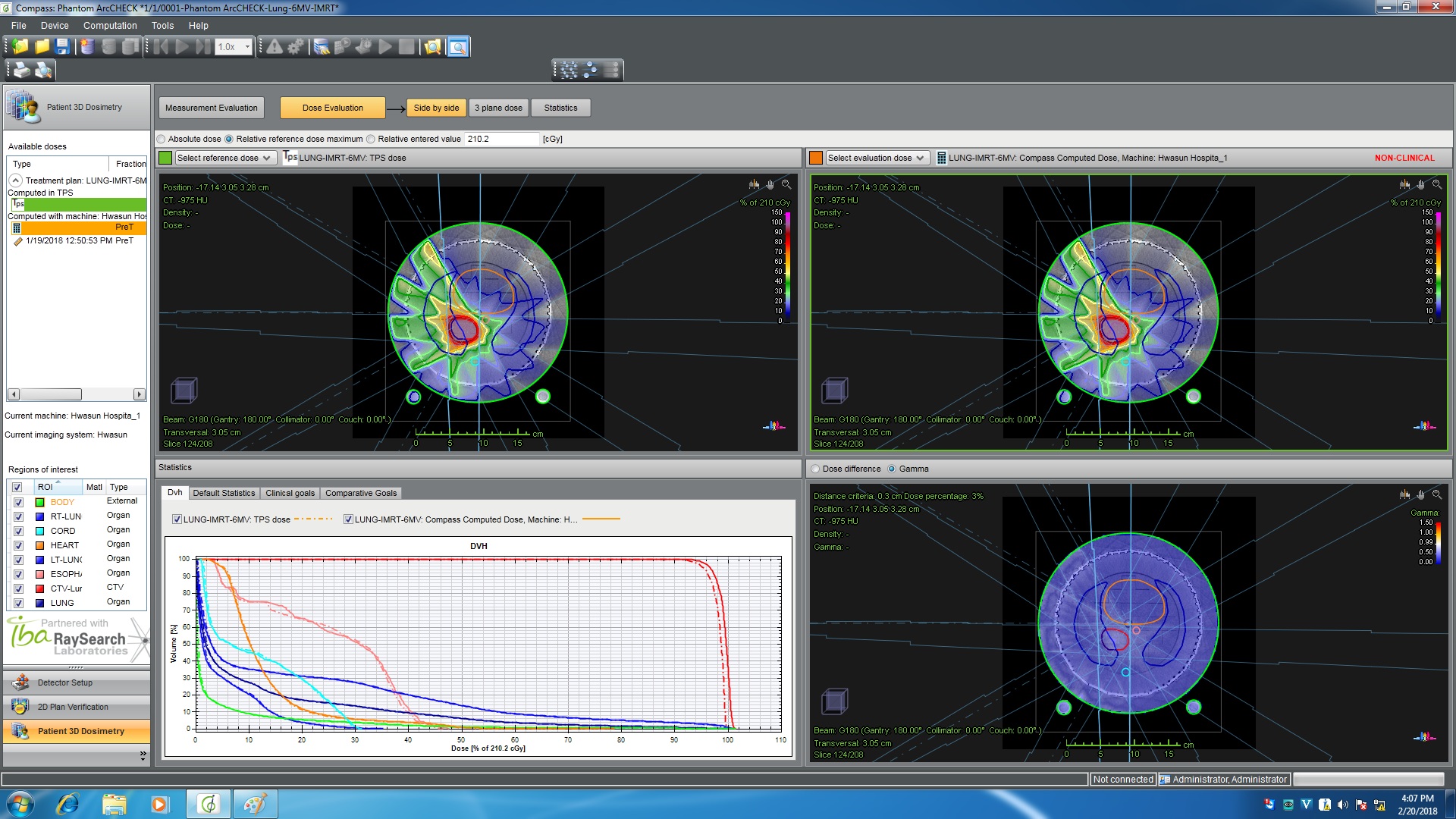

Supplement: S11 Fig — (JPG) [file pone.0209180.s011.jpg]

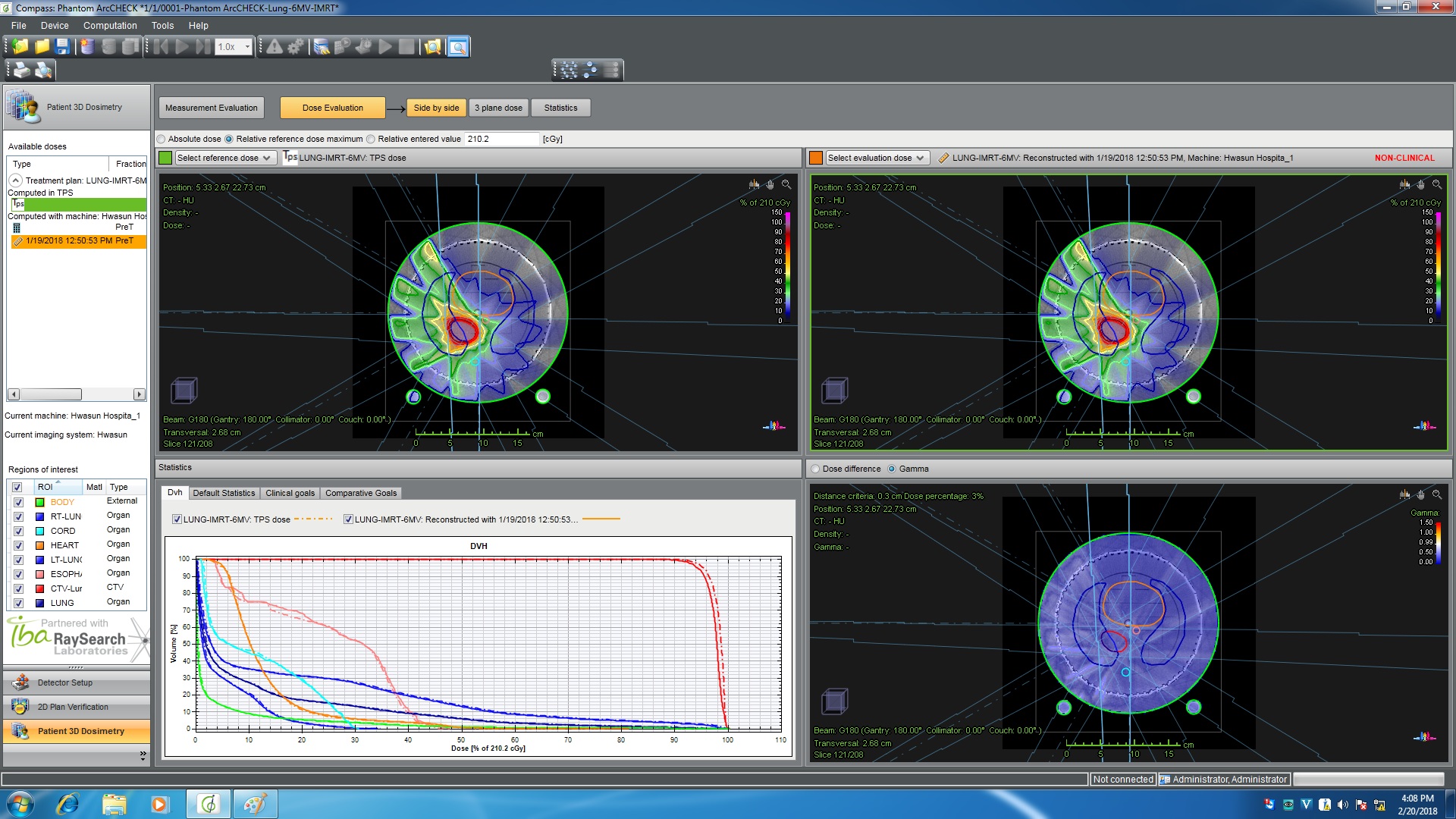

Supplement: S12 Fig — (JPG) [file pone.0209180.s012.jpg]

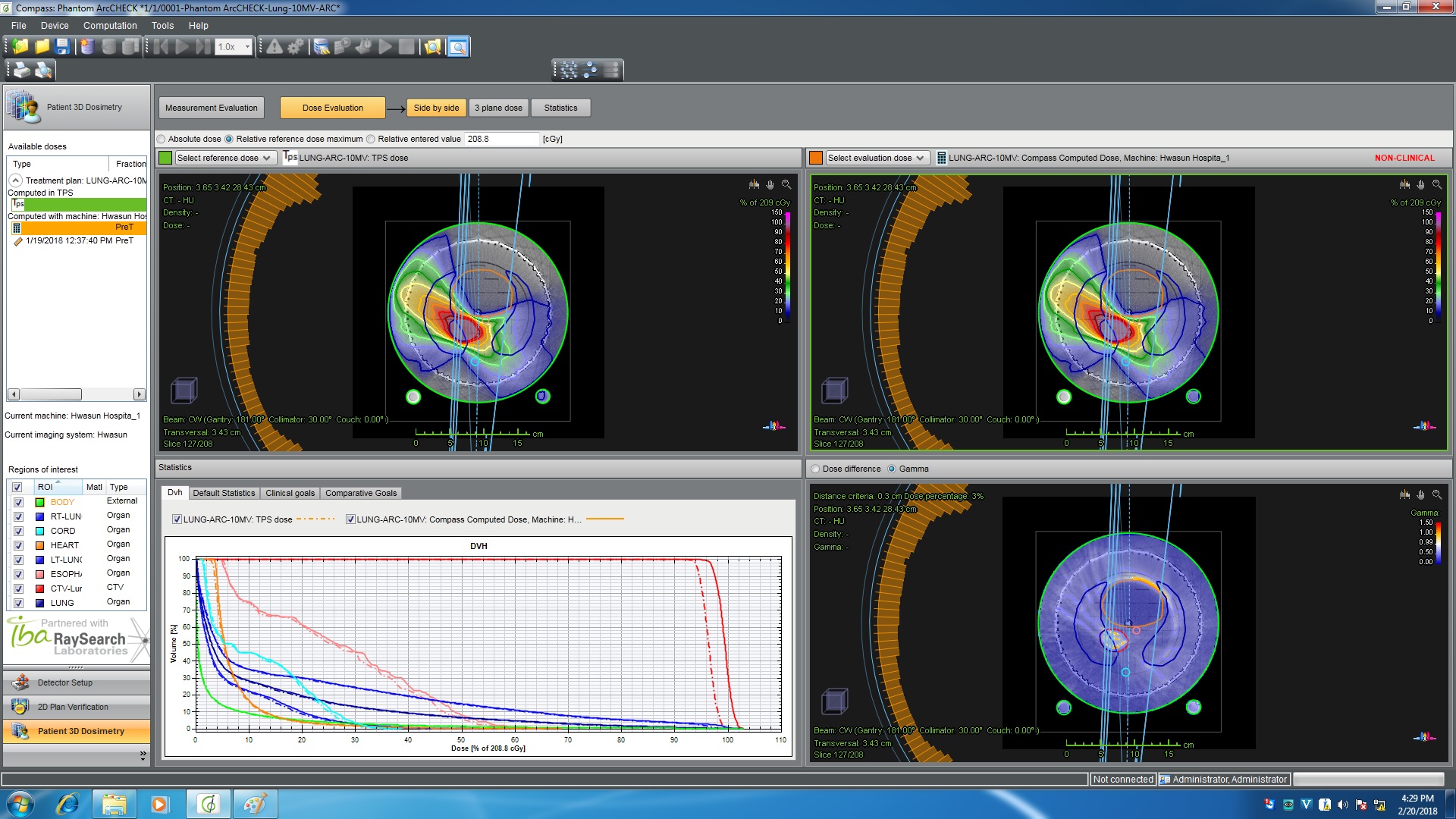

Supplement: S13 Fig — (JPG) [file pone.0209180.s013.jpg]

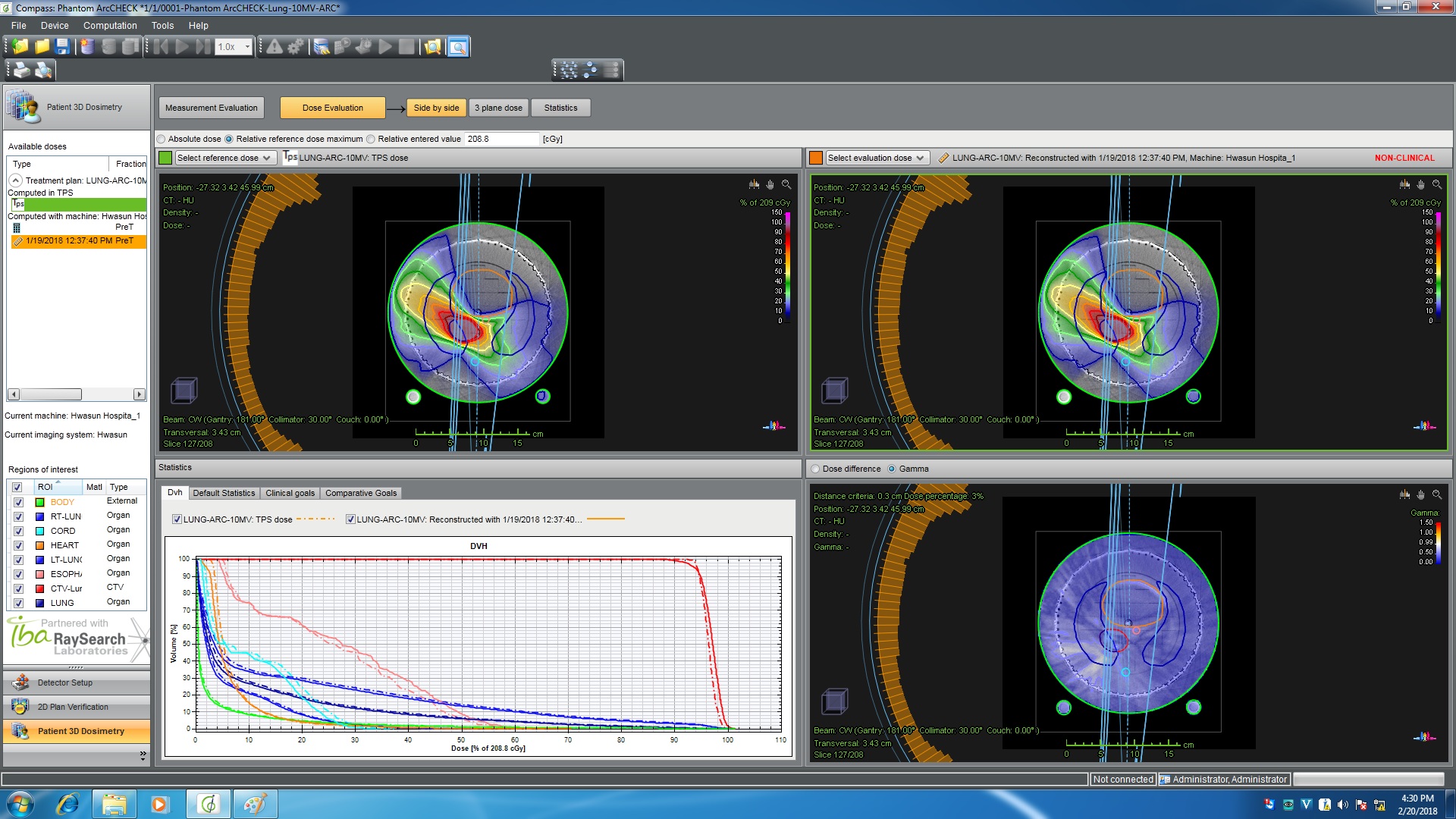

Supplement: S14 Fig — (JPG) [file pone.0209180.s014.jpg]

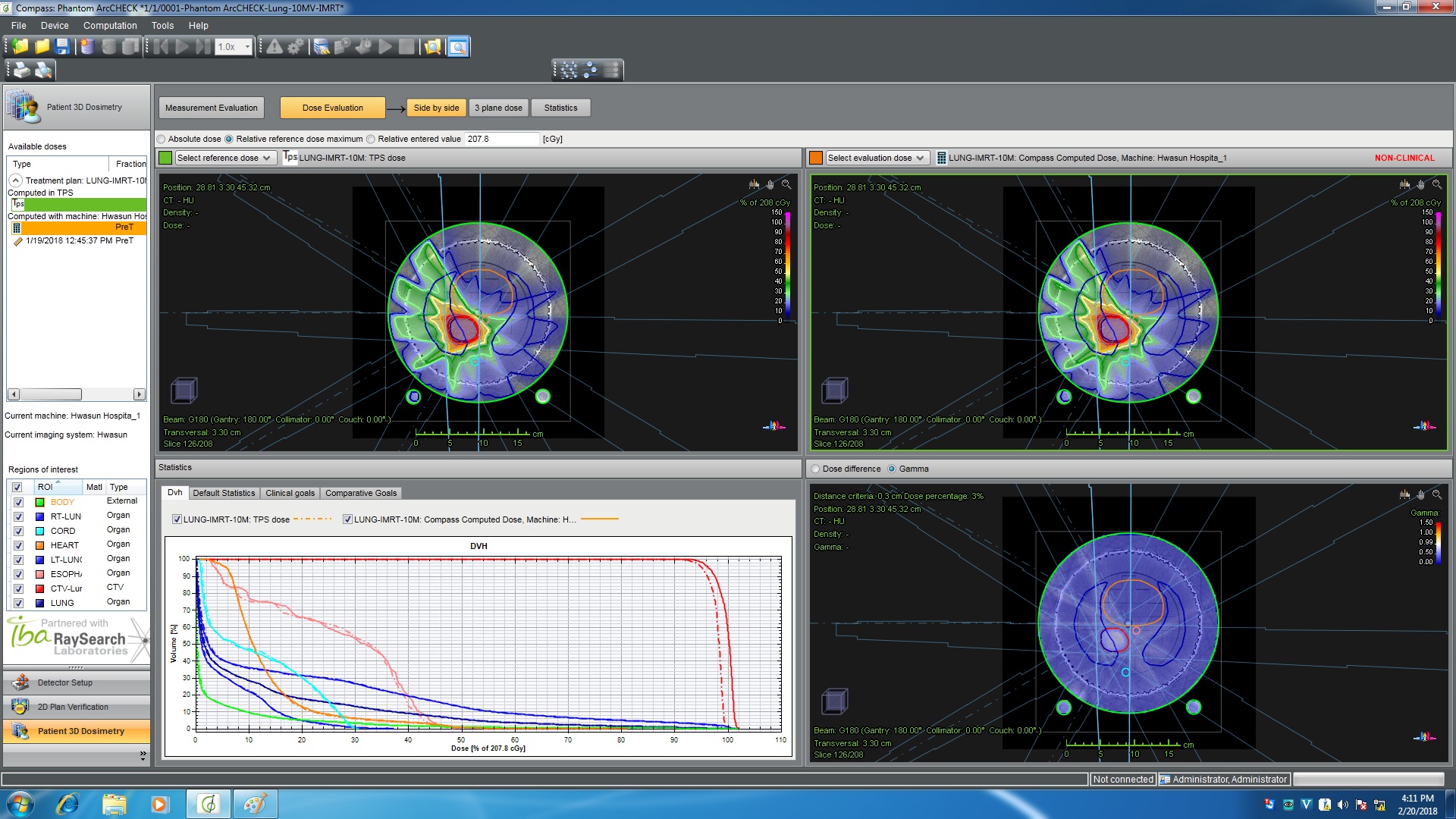

Supplement: S15 Fig — (JPG) [file pone.0209180.s015.jpg]

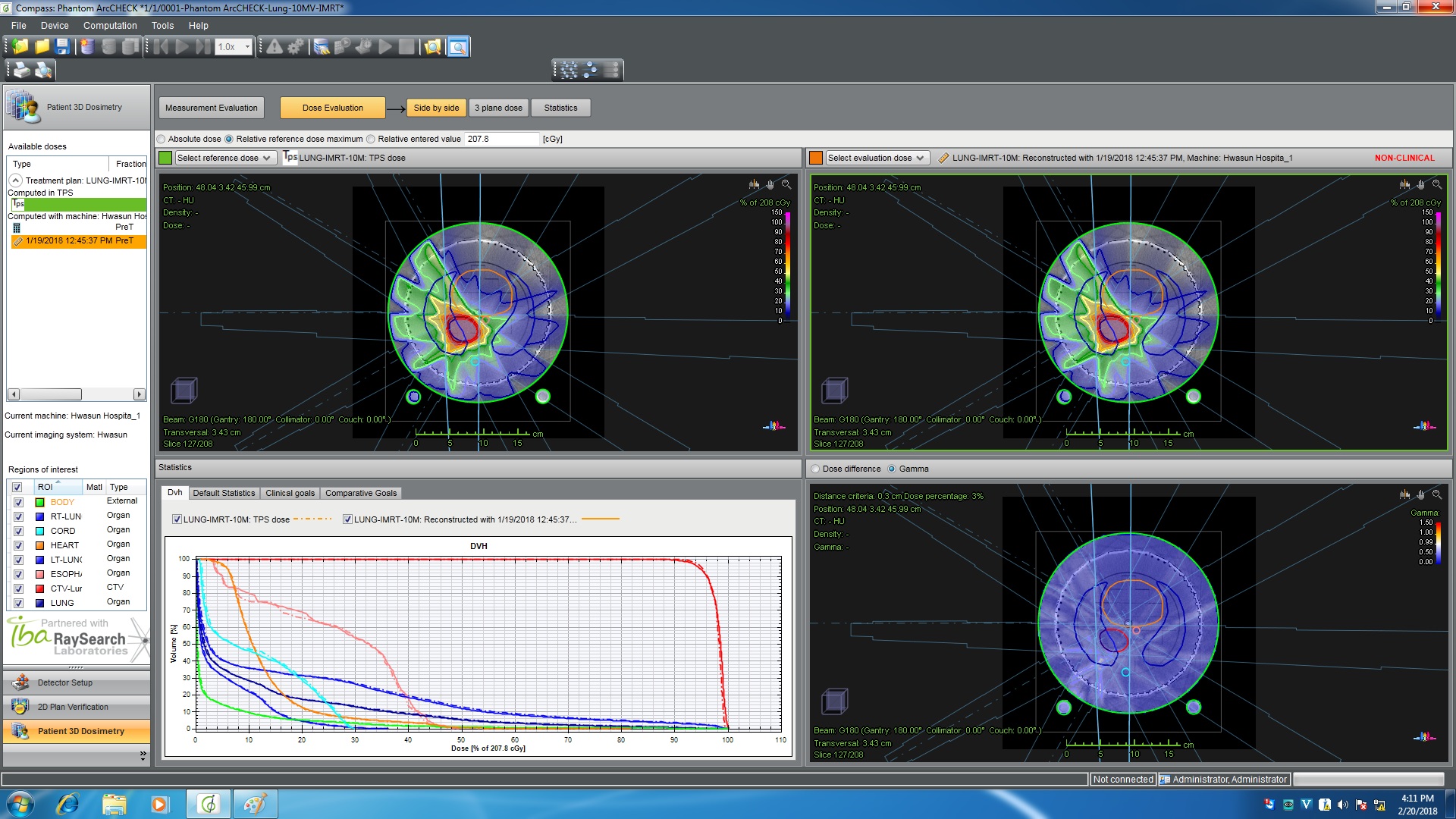

Supplement: S16 Fig — (JPG) [file pone.0209180.s016.jpg]

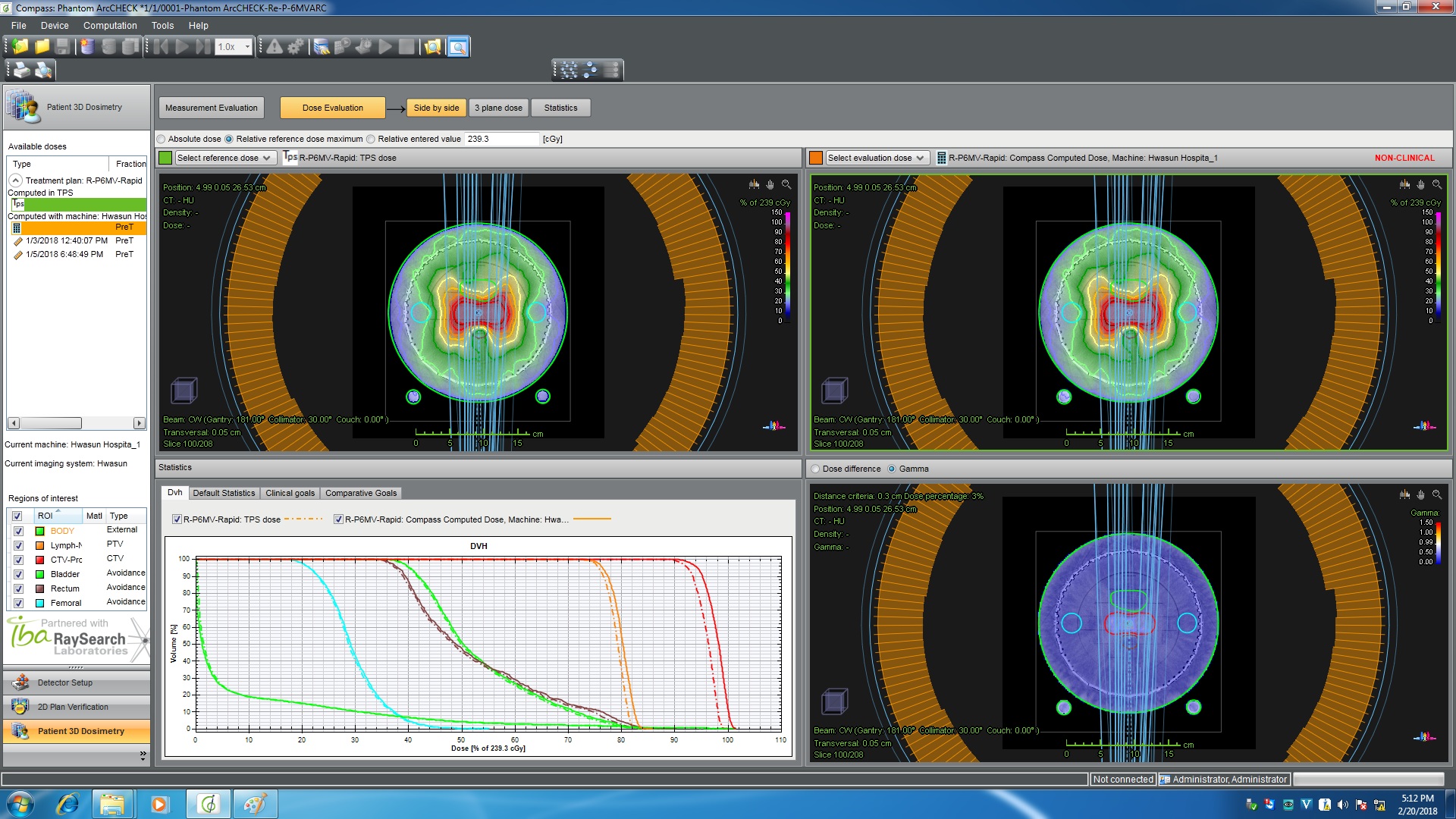

Supplement: S17 Fig — (JPG) [file pone.0209180.s017.jpg]

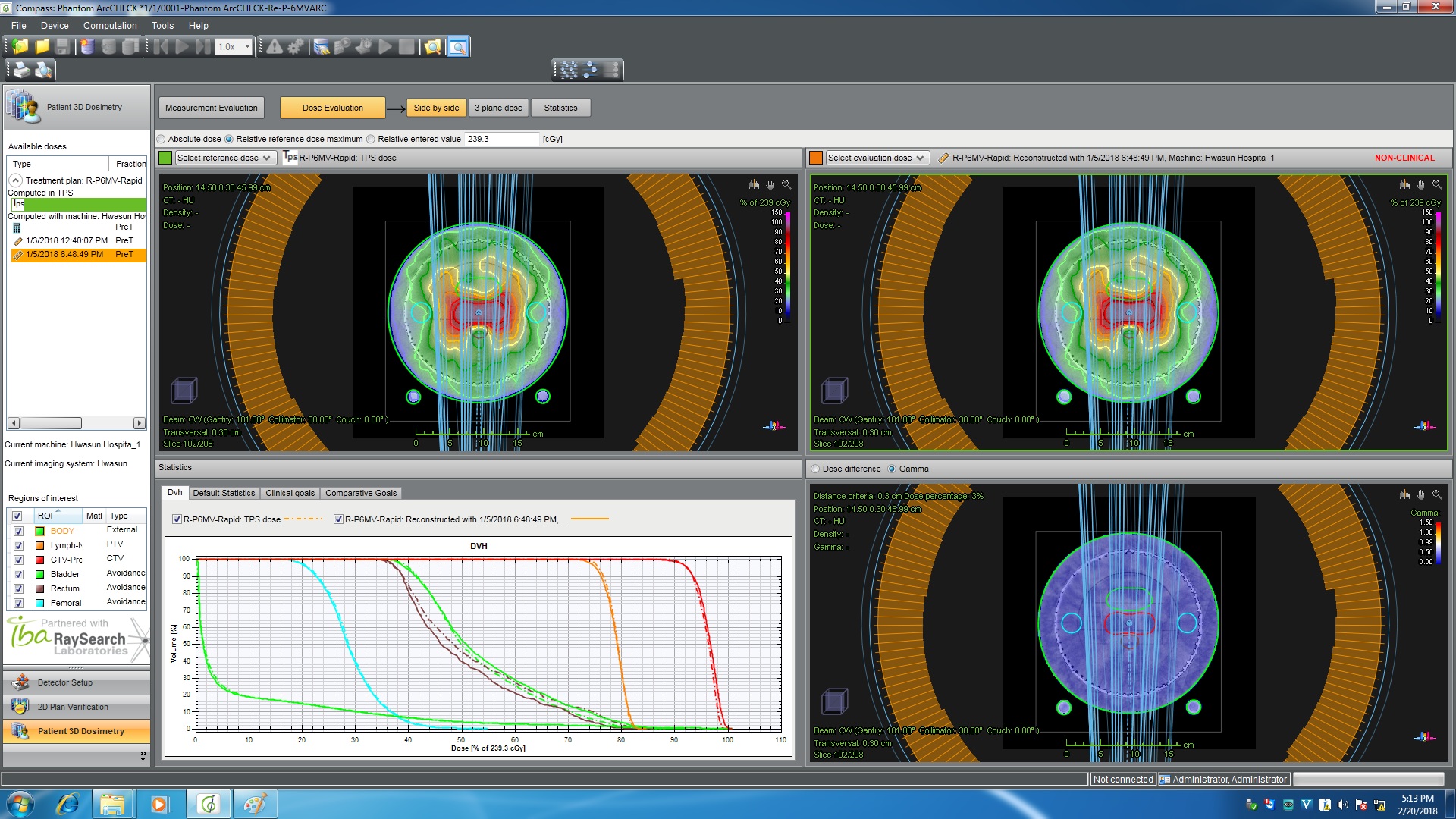

Supplement: S18 Fig — (JPG) [file pone.0209180.s018.jpg]

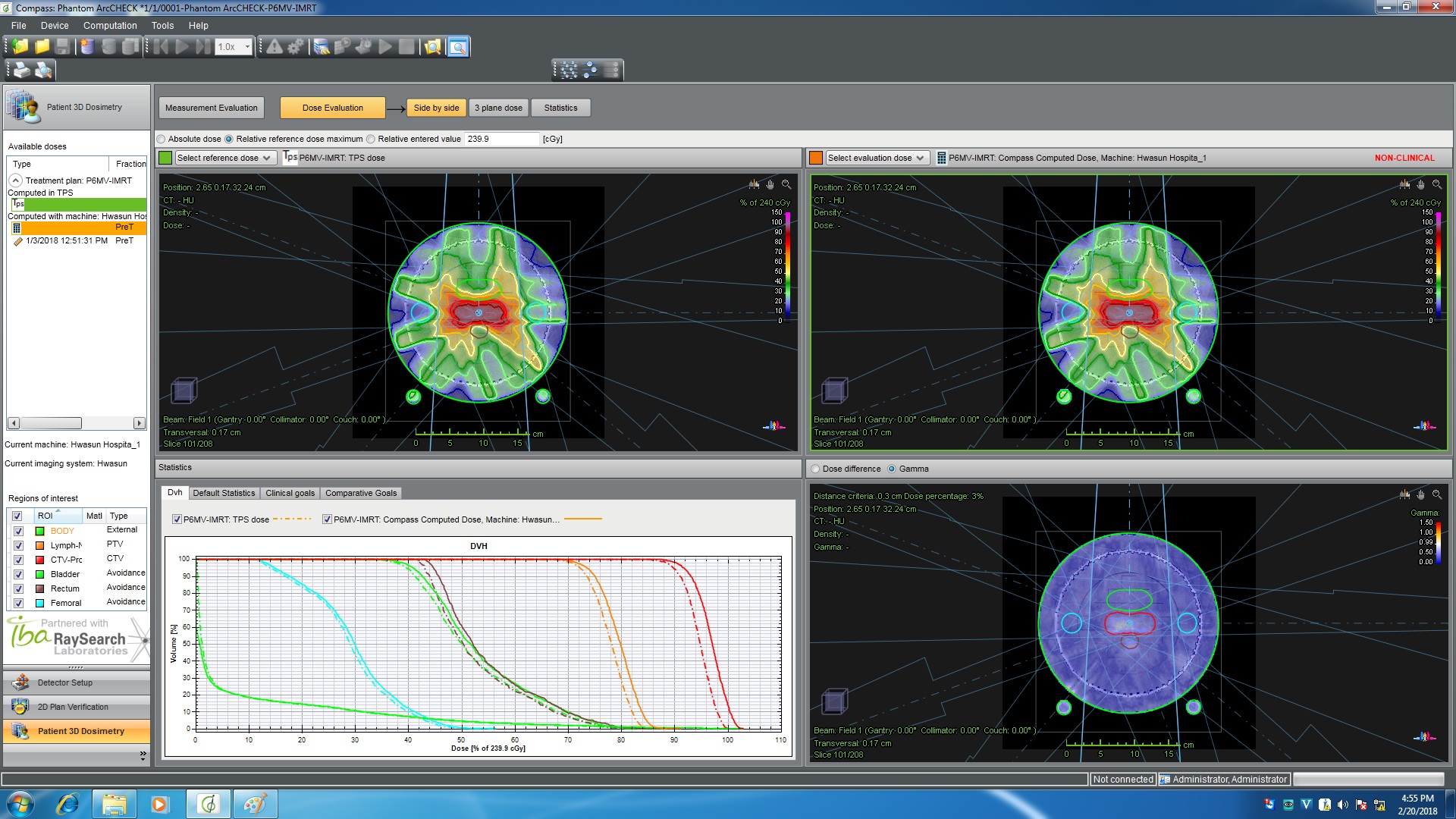

Supplement: S19 Fig — (JPG) [file pone.0209180.s019.jpg]

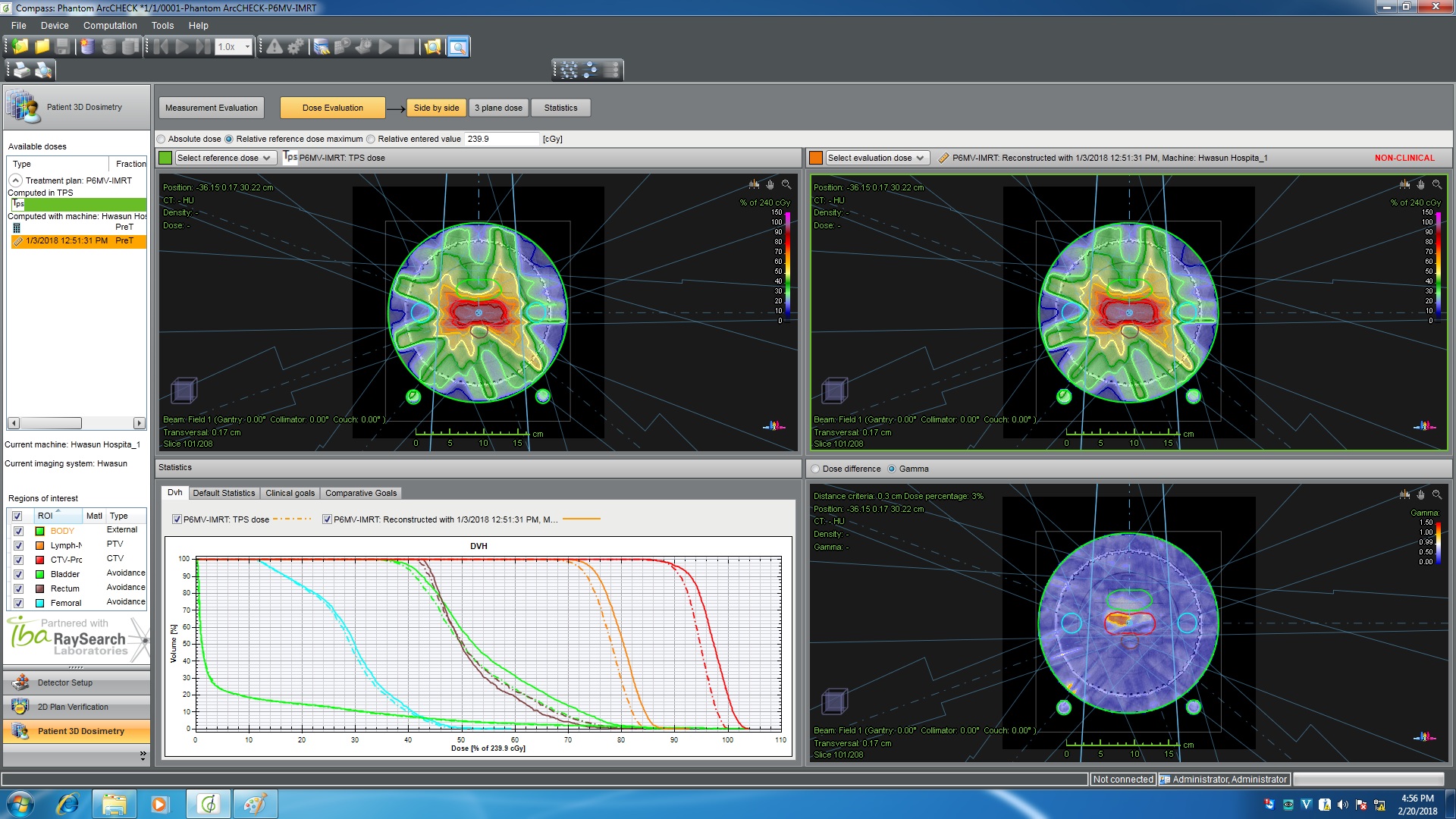

Supplement: S20 Fig — (JPG) [file pone.0209180.s020.jpg]

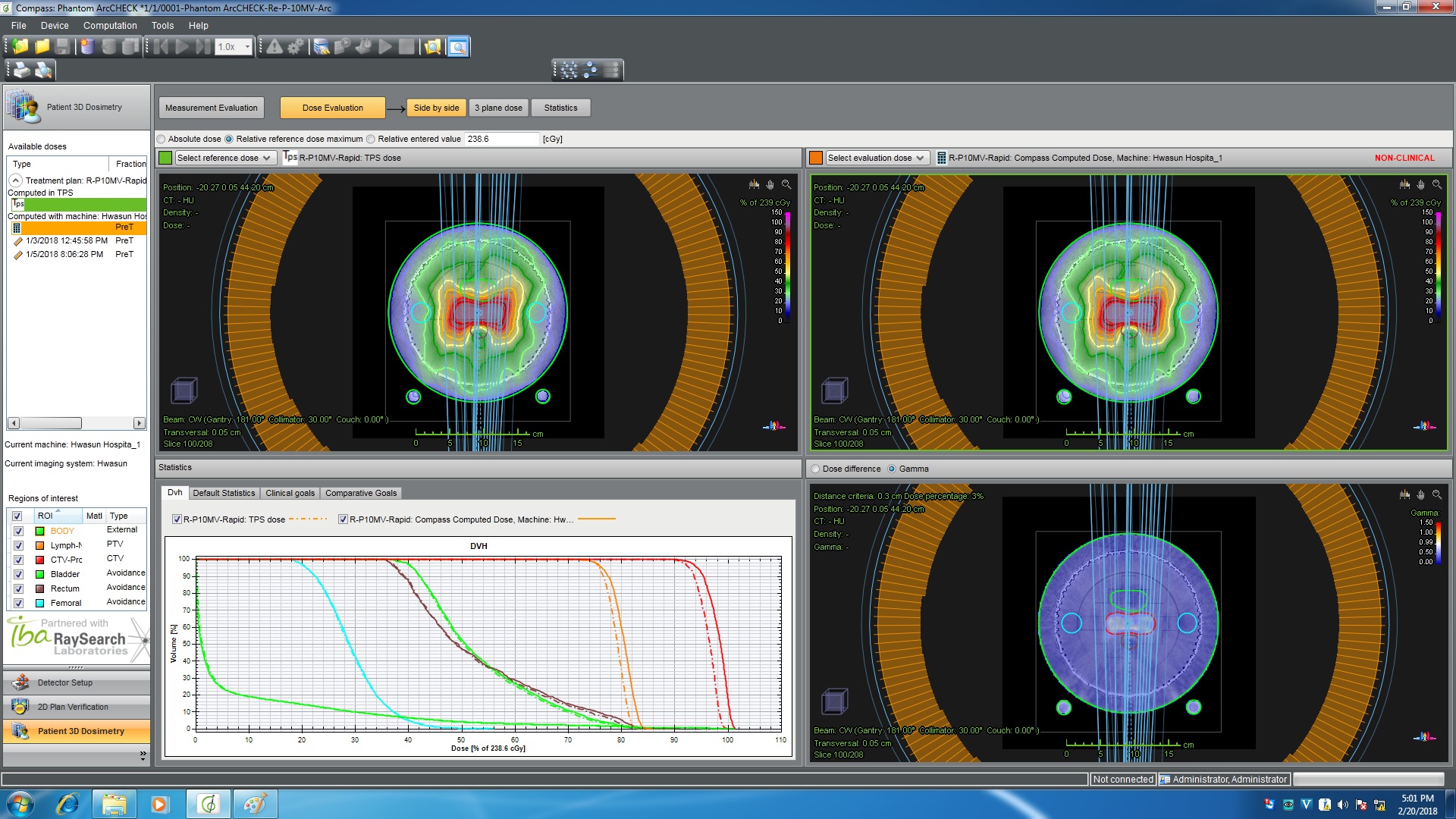

Supplement: S21 Fig — (JPG) [file pone.0209180.s021.jpg]

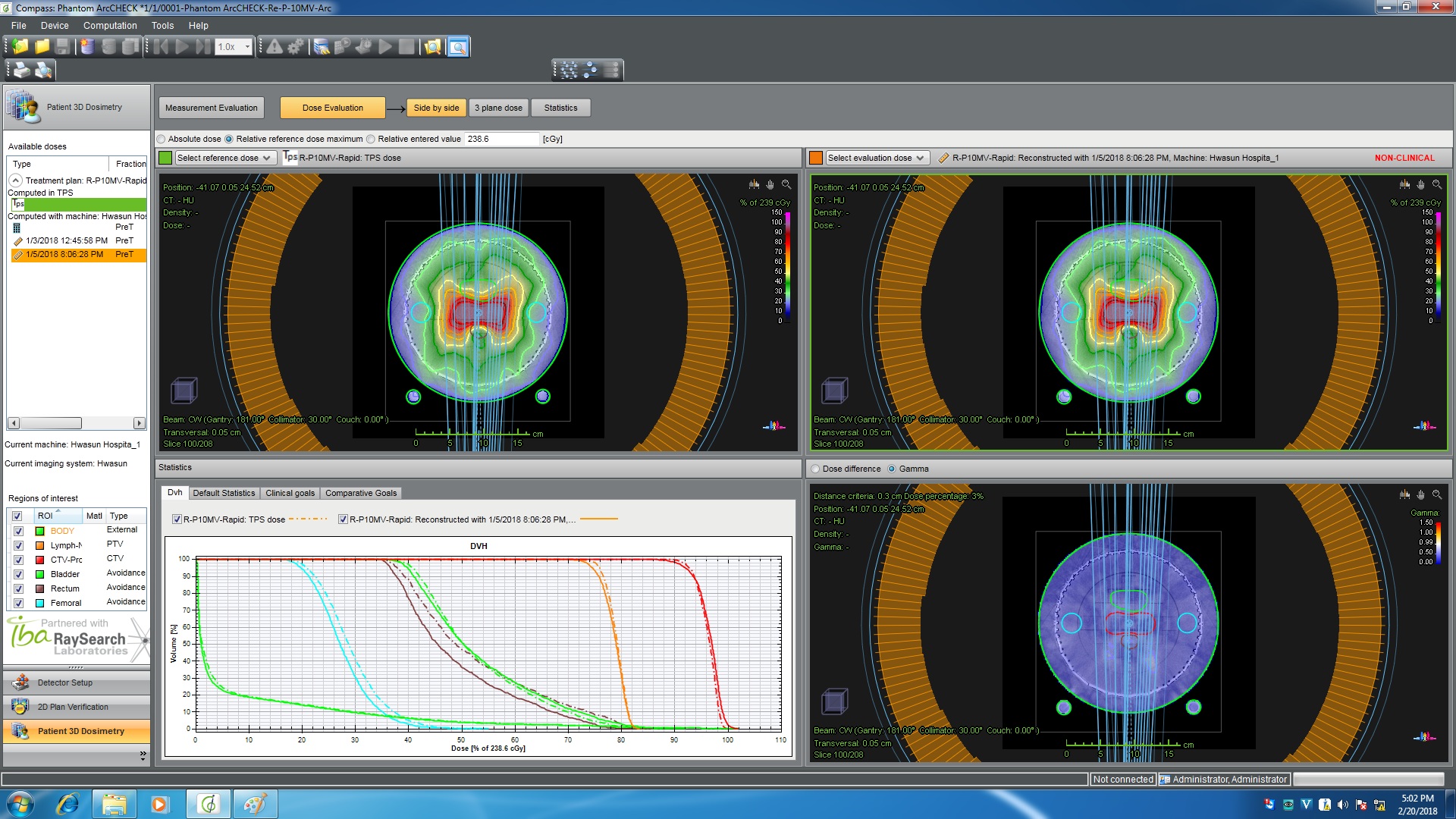

Supplement: S22 Fig — (JPG) [file pone.0209180.s022.jpg]

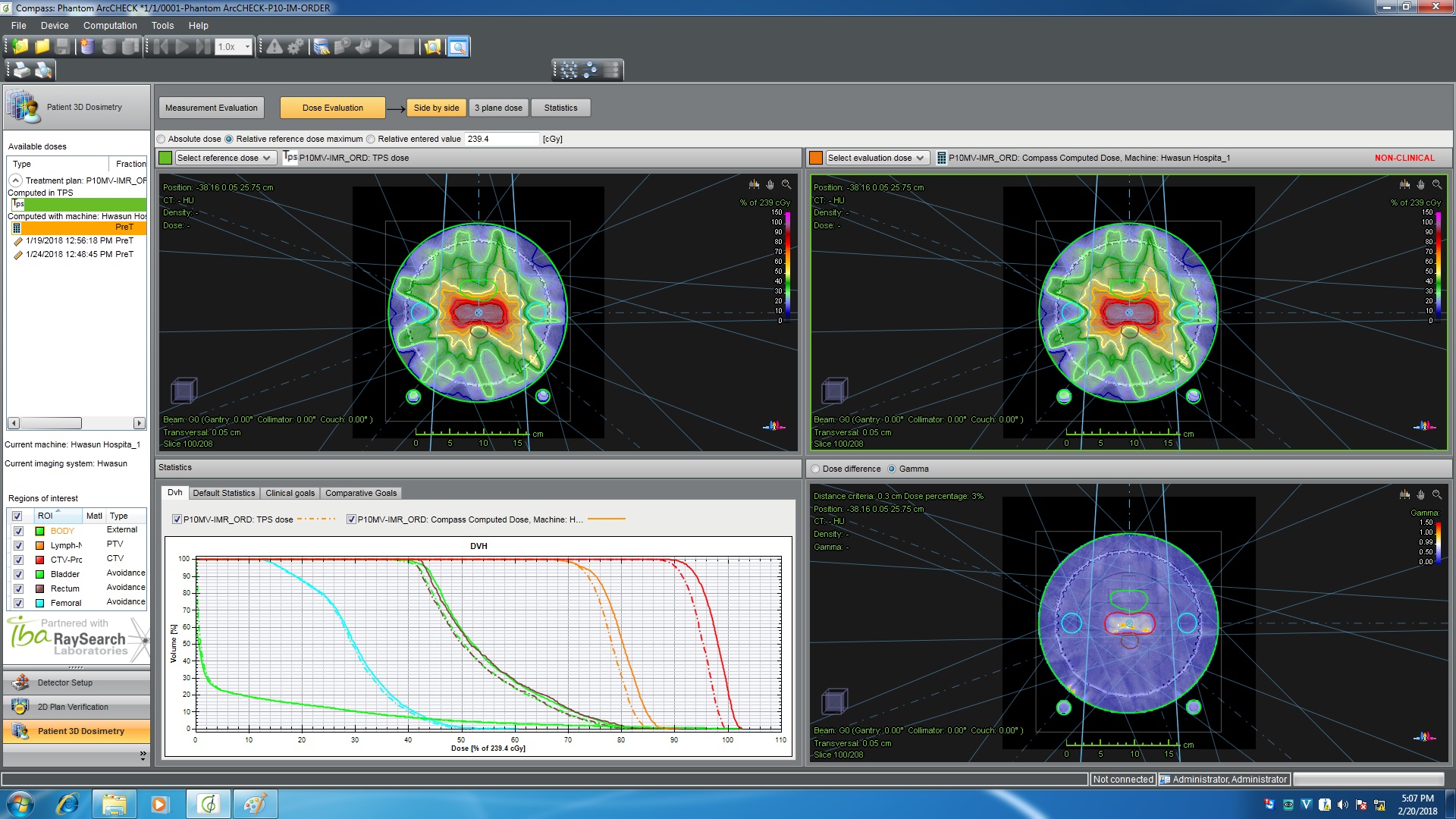

Supplement: S23 Fig — (JPG) [file pone.0209180.s023.jpg]

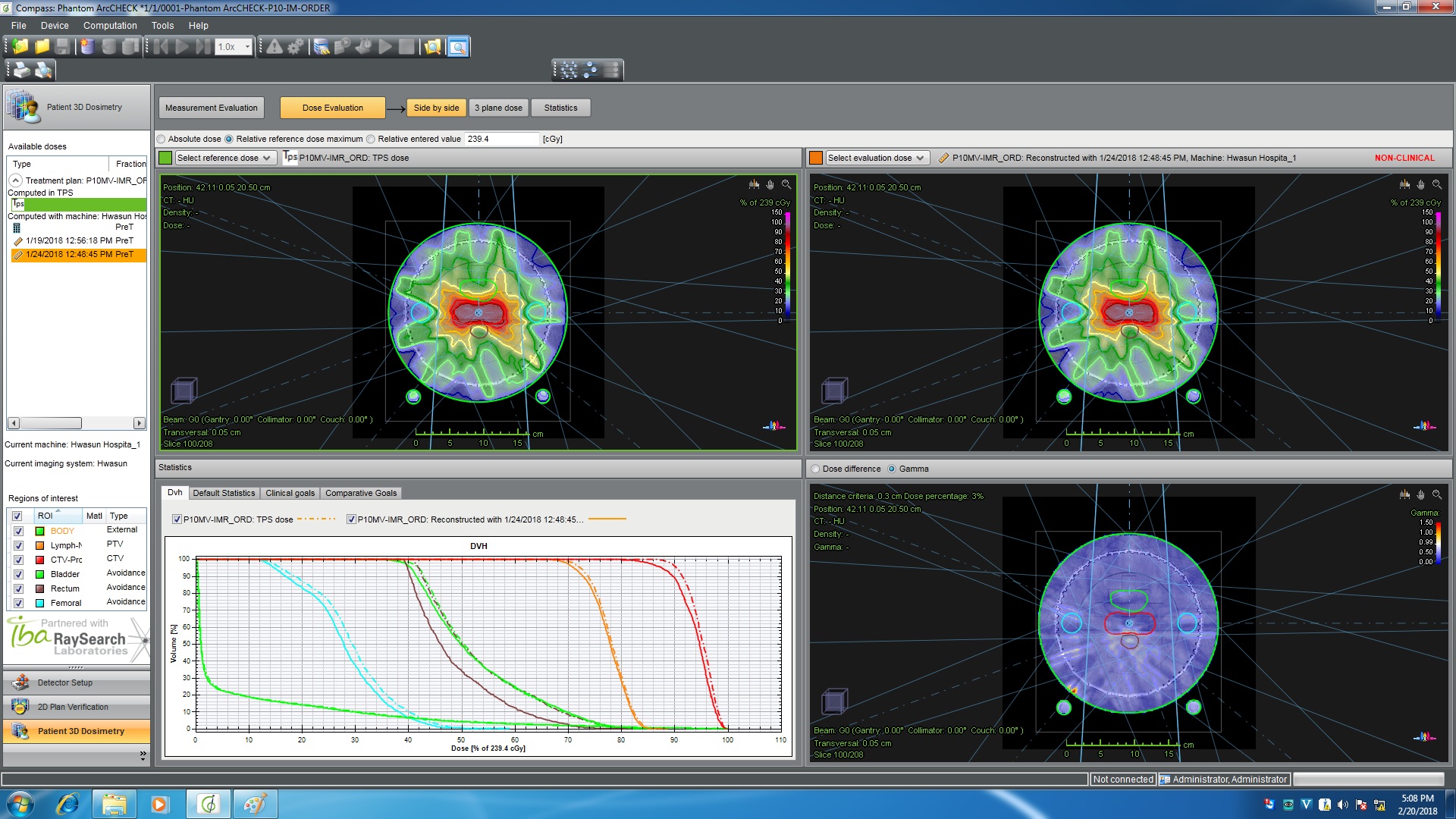

Supplement: S24 Fig — (JPG) [file pone.0209180.s024.jpg]
